# ExPortal and the LiaFSR Regulatory System Coordinate the Response to Cell Membrane Stress in Streptococcus pyogenes

**DOI:** 10.1128/mBio.01804-20

**Published:** 2020-09-15

**Authors:** Yibin Lin, Misu A. Sanson, Luis Alberto Vega, Brittany Shah, Shrijana Regmi, M. Belen Cubria, Anthony R. Flores

**Affiliations:** a Division of Infectious Diseases, Department of Pediatrics, McGovern Medical School, University of Texas Health Sciences Center at Houston, Houston, Texas, USA; b Center for Antimicrobial Resistance and Microbial Genomics, McGovern Medical School, University of Texas Health Sciences Center at Houston, Houston, Texas, USA; Nanyang Technological University

**Keywords:** ExPortal, LiaFSR, cell envelope stress, group A streptococcus, membrane microdomain, two-component regulatory systems

## Abstract

LiaFSR is a gene regulatory system important for response to cell membrane stress in Gram-positive bacteria but is minimally studied in the important human pathogen group A *Streptococcus* (GAS). Using immunofluorescence and immunogold electron microscopy, we discovered that LiaF (a membrane-bound repressor protein) and LiaS (a sensor kinase) reside within the GAS membrane microdomain (ExPortal). Cell envelope stress induced by antimicrobials resulted in ExPortal disruption and activation of the LiaFSR system. The only human antimicrobial peptide whose presence resulted in ExPortal disruption and LiaFSR activation was the alpha-defensin human neutrophil peptide 1 (hNP-1). Elimination of membrane cardiolipin through targeted gene deletion resulted in loss of LiaS colocalization with the GAS ExPortal and activation of LiaFSR, whereas LiaF membrane localization was unaffected. Isogenic mutants lacking either LiaF or LiaS revealed a critical role of LiaF in ExPortal integrity. Thus, LiaF and LiaS colocalize with the GAS ExPortal by distinct mechanisms, further supporting codependence. These are the first data identifying a multicomponent signal system within the ExPortal, thereby providing new insight into bacterial intramembrane signaling in GAS that may serve as a paradigm for Gram-positive bacteria.

## Abstract (precis)

Bacterial two-component systems sense and induce transcriptional changes in response to environmental stressors, including antimicrobials and human antimicrobial peptides. Since the stresses imposed by the host’s defensive responses may act as markers of specific temporal stages of disease progression or host compartments, pathogens often coordinately regulate stress response programs with virulence factor expression. The mechanism by which bacteria recognize these stresses and subsequently induce transcriptional responses remains not well understood. In this study, we showed that LiaFSR senses cell envelope stress through colocalization of LiaF and LiaS with the group A *Streptococcus* (GAS) ExPortal and is activated in direct response to ExPortal disruption by antimicrobials or human antimicrobial peptides. Our studies shed new light on the sensing of cell envelope stress in Gram-positive bacteria and may contribute to the development of therapies targeting these processes.

## INTRODUCTION

The human host has developed an array of defense mechanisms aimed at preventing infection by bacterial pathogens. Antimicrobial peptides (AMPs) represent a first line of defense of the innate immune system against bacterial infection. AMPs are produced at sites of potential pathogen colonization, including skin and mucosal surfaces (e.g., cathelicidins, LL-37), in addition to comprising part of the armamentarium of immune cells (e.g., alpha- and beta-defensins) (1). The mechanisms of action of AMPs differ, but the result is disruption of the bacterial membrane, facilitating killing and clearance (2). In response, many bacterial pathogens have developed a number of strategies to disarm or mitigate the effects of AMPs in order to establish infection (1).

The bacterial membrane is a complex structure critical to maintaining the integrity of the cell. It is now recognized that many bacterial cell processes are maintained in discrete locations within the membrane that are referred to as functional membrane microdomains (FMMs) (3–5). On the basis of the association with the general secretory pathway (6), the microdomain in group A *Streptococcus* (GAS) has been termed the “ExPortal” and, in addition to protein secretion and processing, colocalizes with proteins critical in peptidoglycan precursor synthesis (7). Not surprisingly, given the association with essential cell processes such as cell wall synthesis and division, the bacterial FMM is a key target of host defenses. The naturally occurring lipopeptide daptomycin (DAP) targets FMMs of Gram-positive bacteria, displacing membrane-associated proteins essential for cell wall maintenance (8). Similarly, the human alpha-defensin human neutrophil peptide 1 (hNP-1), restricted to azurophilic granules of neutrophils (9), preferentially disrupts the ExPortal, inhibiting protein secretion and processing (10, 11). The bacterial FMM is an attractive target for the development of novel antimicrobials in the battle against resistance (12). However, much information is lacking regarding FMM content and organization and the downstream effects of antimicrobial-induced FMM disruption.

The ability of bacterial pathogens to colonize a host and grow at the site of infection is dependent upon precise gene regulation in response to external stimuli. To accomplish this feat, many bacteria utilize gene regulatory two-component systems (TCSs) composed of a membrane-bound sensor histidine kinase (HK) that, in response to a stimulus, activates a cognate response regulator (RR) via phosphotransfer (13). Not surprisingly, some bacterial TCSs have been shown to respond to human AMPs (14, 15). The LiaFSR TCS, unique to Gram-positive bacteria, is composed of an HK (LiaS), an RR (LiaR), and an accessory membrane protein of unknown function (LiaF). LiaFSR is a critical component of the cell envelope stress response and is activated following exposure to antimicrobials targeting the cell envelope (e.g., bacitracin [BAC] or daptomycin) or AMPs (e.g., LL-37) (16). Interest in the LiaFSR TCS is rooted in the association between antimicrobial resistance and mutations within the individual TCS components or its downstream effectors (17, 18). While much is known regarding LiaFSR and the development of resistance to antimicrobials, the external host stimuli, mechanisms of signaling, and downstream effects of LiaFSR activation in pathogenic Gram-positive bacteria are largely unresolved.

The HK (LiaS) of the LiaFSR regulatory system is among the members of a specialized class referred to as intramembrane (IM) sensing HKs (IM-HKs). In contrast to HKs that have large extracytoplasmic sensing domains, IM-HKs possess two or more transmembrane domains that, together with accessory membrane proteins (e.g., LiaF), respond to changes in the cell membrane (19). Most of what is known regarding IM-HK activation and signal transduction has been derived from TCSs that respond to antimicrobial-induced stress (e.g., LiaS and BceS) (16, 20). Given the apparent need for accessory proteins and proper IM-HK function, precise subcellular colocalization is assumed but such information for many bacterial pathogens is lacking. Recently, it was shown that the membrane microdomain protein content of Staphylococcus aureus includes IM-HKs such as SaeS and the LiaS homolog VraS (3). High-resolution imaging has also shown subcellular focal interaction between membrane kinases and target regulatory proteins (21, 22). Combined, those studies indicate a critical link between membrane microdomains and signaling pathways.

Here, we begin to define the relationship between LiaFSR and the membrane microdomain (ExPortal) in GAS by testing the hypothesis that signal transduction through LiaFSR is directly linked to the GAS ExPortal. We discovered that LiaF and LiaS colocalize with the GAS ExPortal and respond to antimicrobial and human AMP-induced membrane stress. In addition, we have uncovered a critical role of cardiolipin (CL) in LiaS localization and of the accessory protein LiaF in GAS ExPortal integrity. Given the critical role of the membrane microdomain and high conservation of LiaFSR, our findings may serve as an archetype for multiple Gram-positive pathogens.

## RESULTS

### LiaF and LiaS colocalize with the ExPortal.

**FIG 1:**
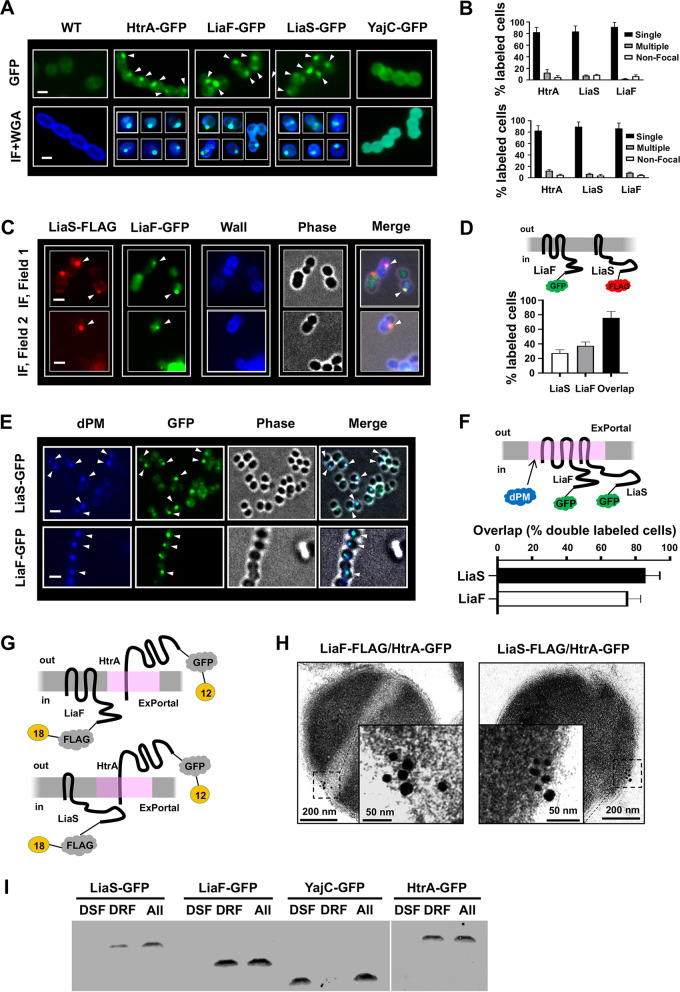
LiaF and LiaS exhibit focal localization and are coincident with the ExPortal. (A) GFP-tagged proteins in the GAS membrane were detected by fluorescence microscopy (top row) and immunofluorescence (IF) microscopy (bottom row). All IF images were prepared as described in Materials and Methods. The bottom row shows images merged from two fluorescence channels (IF+WGA). Arrowheads indicate GFP-tagged proteins. Scale bars, 1 μm. WGA, wheat germ agglutinin. (B) The distribution of HtrA-GFP, LiaS-GFP, and LiaF-GFP was manually quantified by fluorescence microscopy (upper panel) and IF (lower panel) as described for panel A. Data are shown as means ± standard deviations (SD) of results from a minimum of 300 stained cells from three independent experiments. (C) GAS cells expressing LiaF-GFP and LiaS-FLAG were immunostained as described in Materials and Methods. Images were merged with a phase-contrast image as indicated above the individual panels. The upper panel and lower panel show separate images from independent representative fields. Arrowheads indicate focal signal of LiaF-GFP and LiaS-FLAG. Scale bars, 1 μm. (D) (Upper panel) Schematic of LiaF-GFP and LiaS-FLAG variants used for immunodetection. (Lower panel) Colocalization of LiaF-GFP with LiaS-FLAG was quantified by immunofluorescence as described for panel C. Data shown are means ± SD of results from a minimum of 300 stained cells from three independent experiments. (E) GAS strains expressing LiaS-GFP (upper panels) and LiaF-GFP (lower panels) were stained with dansyl-polymyxin B (dPM) as described in Materials and Methods. Images were merged with a phase-contrast image as indicated above the individual panels. Scale bars, 1 μm. (F) (Upper panel) Double-labeling schematic to detect protein colocalization with the ExPortal. (Lower panel) Results of colocalization of LiaF-GFP and LiaS-GFP with ExPortal are quantified by fluorescence as described for panel E. Data shown are means ± SD of results from a minimum of 300 stained cells from three independent experiments. (G) Immunogold labeling schematic for LiaF/LiaS-FLAG and HtrA-GFP. (H) Colocalization of LiaF/LiaS and ExPortal was analyzed by transmission electron microscopy. Strains expressing LiaF-FLAG (left panel) or LiaS-FLAG (right panel) and HtrA-GFP were subjected to immunogold electron microscopy and stained to detect GFP (12-nm-diameter beads) and FLAG (18-nm-diameter beads). (I) Immunoblot to detect LiaS and LiaF in the ExPortal membrane fractions (DRF). Membrane fractions were prepared as described in Materials and Methods. LiaS-GFP, LiaF-GFP, YajC-GFP, and HtrA-GFP were identified by Western blotting using an anti-GFP antibody. DSF, detergent-sensitive fraction; DRF, detergent-resistant fraction; All, total membrane fractions.

Inasmuch as the antimicrobials and AMPs have been independently noted to affect the GAS ExPortal and LiaFSR, we hypothesized that LiaFSR signal transduction may be linked to ExPortal disruption. To begin investigating the association between LiaFSR and the ExPortal, we constructed isogenic green fluorescent protein (GFP)-tagged or FLAG-tagged strains (7, 23) with the proteins expressed at their respective native chromosomal loci in the common *emm3* background (wild type [WT], MGAS10870) (see Table S1 in the supplemental material). Isogenic, GFP/FLAG-tagged strains showed identical growth characteristics (see Fig. S1 in the supplemental material) and exhibited no differences in cell morphology as observed by phase-contrast microscopy compared to the parental strain (data not shown). We hypothesized that membrane-bound LiaS (HK) and LiaF (accessory protein) colocalize within the ExPortal. Consistent with previous studies showing that the protease HtrA was a component of and indicator for the GAS ExPortal (10) (Fig. S2; see also Fig. S3), GAS cells with HtrA-GFP demonstrated single points of focal localization by fluorescence microscopy (Fig. 1A and B, upper panels). Similarly, GAS cells with LiaF-GFP or LiaS-GFP exhibited single foci (Fig. 1A and B, upper panels). The single focal pattern was confirmed by immunofluorescence (IF) microscopy (Fig. 1A and B, lower panels). The focal localization of HtrA, LiaS, and LiaF contrasted with that of YajC, which, consistent with previous examination (7, 10), demonstrated a uniform circumferential GAS cell distribution (Fig. 1A). Finally, since it was reported previously that fusion to a fluorescent partner can alter the localization patterns of certain proteins (24), the localizations of LiaF and LiaS were also examined following fusion to a small epitope tag (FLAG) (7). In a manner identical to that seen with their GFP-tagged counterparts, GAS cells with LiaF-FLAG or LiaS-FLAG localized to unique focal sites (Fig. S4B and C), in contrast to the results seen with a FLAG-tagged version of YajC (Fig. S4D). Overall, the data suggest that LiaF and LiaS are found in single membrane foci similarly to proteins known to colocalize with the ExPortal.

10.1128/mBio.01804-20.1 FIG S1 Growth curves of GFP-tagged proteins in GAS. Download FIG S1, DOCX file, 0.3 MB. Copyright © 2020 Lin et al. 2020 Lin et al. This content is distributed under the terms of the Creative Commons Attribution 4.0 International license.

10.1128/mBio.01804-20.2 FIG S2 Dansyl-polymyxin B treatment does not alter membrane ExPortal profile. Download FIG S2, DOCX file, 0.8 MB. Copyright © 2020 Lin et al. 2020 Lin et al. This content is distributed under the terms of the Creative Commons Attribution 4.0 International license.

10.1128/mBio.01804-20.3 FIG S3 HtrA colocalizes with the ExPortal. Download FIG S3, DOCX file, 0.4 MB. Copyright © 2020 Lin et al. 2020 Lin et al. This content is distributed under the terms of the Creative Commons Attribution 4.0 International license.

10.1128/mBio.01804-20.4 FIG S4 Focal localization of LiaS-FLAG and LiaF-FLAG. Download FIG S4, DOCX file, 0.2 MB. Copyright © 2020 Lin et al. 2020 Lin et al. This content is distributed under the terms of the Creative Commons Attribution 4.0 International license.

10.1128/mBio.01804-20.8 TABLE S1 Bacterial strains used in this study. Download Table S1, DOCX file, 0.02 MB. Copyright © 2020 Lin et al. 2020 Lin et al. This content is distributed under the terms of the Creative Commons Attribution 4.0 International license.

To further investigate LiaF and LiaS cell membrane distribution, we used IF microscopy to simultaneously examine the localization patterns of GAS cells containing both LiaF-GFP and LiaS-FLAG. Merged images demonstrate overlap of LiaF with LiaS fluorescence consistent with identical locations within the cell membrane (Fig. 1C and D). Using dansyl-polymyxin B (dPM), previously shown to specifically target the ExPortal (11), and GFP fluorescence, LiaF-GFP and LiaS-GFP were demonstrated to reside within the GAS ExPortal (Fig. 1E and F). Importantly, the concentrations of dPM required for visualization did not alter ExPortal integrity (Fig. S2A and B). The ExPortal has previously been shown to be critical for secretion of the GAS cysteine protease SpeB (10). Thus, we also assessed ExPortal function following dPM exposure and observed no differences in secreted SpeB activity from that seen with untreated cells (Fig. S2C). LiaR has previously been shown to directly regulate the gene encoding the RNA polymerase-binding protein SpxA in S. mutans (25) (SpxA2 in GAS) and has been used as an indicator of LiaFSR activation in GAS (26). Thus, to ensure that chimeric LiaF and LiaS proteins remained functional and that dPM did not alter LiaFSR activation, we assayed *spxA2* transcript levels following dPM exposure and observed no significant transcript level differences relative to the untreated cells (Fig. S2D). GAS cell membrane location of LiaF and LiaS was compared with that of ExPortal resident protein HtrA (Fig. S3) by immunogold electron microscopy. Immunogold staining using beads of different sizes confirmed that LiaF and LiaS colocalize to the same site as HtrA (10) (Fig. 1G and H). Finally, we extracted the GAS ExPortal membrane fraction (the detergent-resistant fraction [DRF]) and, using immunoblotting with anti-GFP antibody, demonstrated that GFP-tagged LiaS, LiaF, and HtrA resided within the ExPortal (Fig. 1I). In summary, the data confirm that LiaF and LiaS reside within the same space as the ExPortal in GAS.

### Antimicrobials disrupt LiaF, LiaS, and ExPortal colocalization and activate LiaFSR.

**FIG 2:**
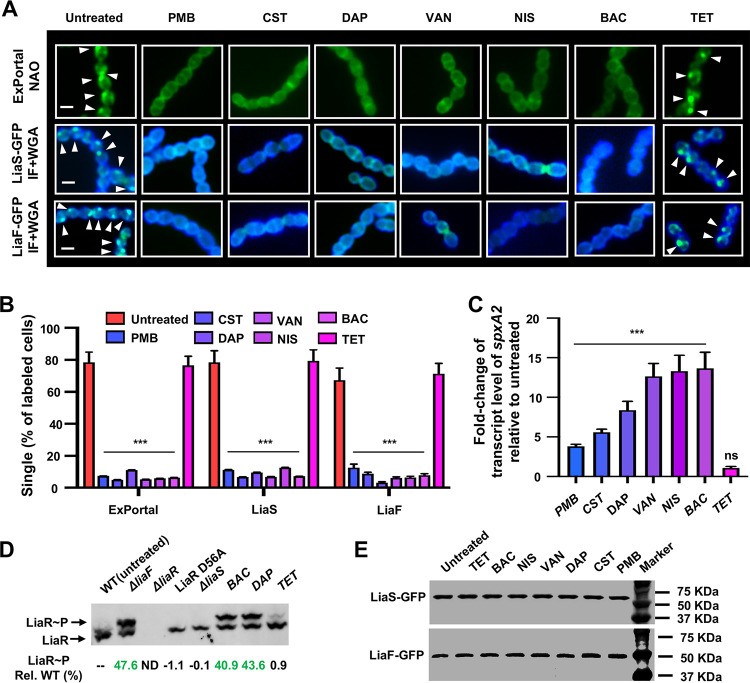
Antimicrobials disrupt LiaF, LiaS, and ExPortal colocalization patterns and activate LiaFSR 3CS. (A) The effect of antimicrobials on the distribution of ExPortal, LiaS-GFP, and LiaF-GFP was quantitated by fluorescence microscopy (ExPortal, NAO staining) and immunofluorescence (LiaS-GFP and LiaF-GFP). GAS cells were treated with PMB (50 μg/ml), CST (50 μg/ml), DAP (0.4 μg/ml), VAN (0.5 μg/ml), NIS (4 μg/ml), BAC (1 μg/ml), or TET (1 μg/ml) as described in Materials and Methods. (Top row) Cells treated with antibiotics as indicated on the top of the panel were stained with NAO and detected by fluorescence microscopy. (Middle and bottom rows) GAS expressing LiaF-GFP or LiaS-GFP were immunostained as described in Materials and Methods. The middle row (LiaS) and bottom row (LiaF) show images merged from two fluorescence channels (IF+WGA). Arrowheads indicate ExPortal or GFP-tagged proteins. Scale bars, 1 μm. (B) Manual quantification of single points of staining of cells treated as described in the panel A legend. Data shown are means ± SD of results from a minimum of 300 stained cells from three independent experiments. (C) The effect of antimicrobial treatment on the activation of LiaFSR 3CS was assessed by the transcription level of *spxA2*. The gene-transcript levels of *spxA2* were quantitated (relative to an endogenous control, *tufA*) following treatment using the indicated antimicrobial as described for panels A and B and compared to those quantitated for the untreated sample. ns, not significant. (D) Determination of LiaR phosphorylation (LiaR∼P) level using Phos-tag gel. Samples were harvested at a time identical to the assessments described for panels A to D. LiaR∼P levels (percentage relative to the WT, untreated) are indicated below the individual lanes. The LiaR-D56A strain was unable to be phosphorylated due to a mutation of the conserved/essential aspartate residue. Similarly, LiaR exists in an unphosphorylated state in the absence of LiaS (Δ*liaS*). ND, not done. (E) The effect of antimicrobials on the LiaF and LiaS cellular protein levels. After exposure to antimicrobial agents as described for panel A, LiaF-GFP and LiaS-GFP were monitored by Western blotting using an anti-GFP antibody. *P* values were determined by Student's *t* test. ***, *P < *0.001 relative to untreated. Abbreviations: BAC, bacitracin; CST, colistin; DAP, daptomycin; NIS, nisin; PMB, polymyxin B; TET, tetracycline; VAN, vancomycin.

Previous work has shown that disruption of the ExPortal by AMPs resulted in redistribution of ExPortal-associated proteins such as SecA and HtrA (11). We hypothesized that activation of LiaFSR is triggered by disruption of the ExPortal by antimicrobials or AMPs. Data on LiaFSR from Bacillus subtilis suggest that LiaF may act as an inhibitory protein with respect to LiaS (27). Also, antimicrobials that target the bacterial cell membrane disrupt FMMs such as the GAS ExPortal (11). Thus, we examined whether antimicrobials that disrupt ExPortal organization also affect LiaF and LiaS colocalization and subsequent LiaFSR activation. MGAS10870 was grown to mid-exponential phase and treated with subinhibitory concentrations of six commonly used antimicrobials. GAS cells were harvested and examined for ExPortal integrity using nonyl acridine orange (NAO) staining and for LiaFSR activation using targeted *spxA2* transcript level measurements. No significant differences in growth compared to untreated cells were observed for the concentration of antimicrobials used in the assay (Fig. S5). The typical focal NAO staining pattern of the ExPortal was disrupted by treatment with cell membrane or cell wall-active antimicrobial agents but not by treatment with tetracycline (protein synthesis inhibitor) (Fig. 2A and B). Treatment also resulted in the concomitant redistribution of LiaF and LiaS from single foci to cells with multiple foci or a diffuse pattern (Fig. 2A and B), indicating a dependence on ExPortal integrity. Compared to untreated cells, we observed significant increases in *spxA2* transcript levels following the addition of cell wall-active antimicrobials (Fig. 2C). In addition, as a more direct measure of LiaFSR activation, we assayed LiaR phosphorylation levels using Phos-tag following bacitracin (BAC) or daptomycin (DAP) treatment. We observed significantly increased LiaR phosphorylation following antimicrobial treatment using cell envelope-active compounds, confirming LiaFSR activation (Fig. 2D). The level of LiaR phosphorylation observed following antimicrobial treatment was similar to that seen with an isogenic mutant lacking LiaF (Δ*liaF*), confirming the inhibitory role of LiaF in the LiaFSR system. Further, activation was specific to cell envelope-active antimicrobials and did not represent a general stress response as no activation was seen with tetracycline—similarly to strains lacking the HK (Δ*liaS*) or mutated at the essential aspartate residue of LiaR (LiaR-D56A) (Fig. 2D). Treatment with antimicrobials did not result in LiaF or LiaS degradation (Fig. 2E). Overall, the data show that disruption of the ExPortal results in the redistribution of LiaF and LiaS leading to activation and are consistent with a model where LiaF acts as an inhibitor of LiaS activity.

10.1128/mBio.01804-20.5 FIG S5 CFU enumeration following growth in the presence of antimicrobials. Download FIG S5, DOCX file, 0.2 MB. Copyright © 2020 Lin et al. 2020 Lin et al. This content is distributed under the terms of the Creative Commons Attribution 4.0 International license.

### hNP-1 disrupts LiaF and LiaS colocalization and activates LiaFSR 3CS.

**FIG 3:**
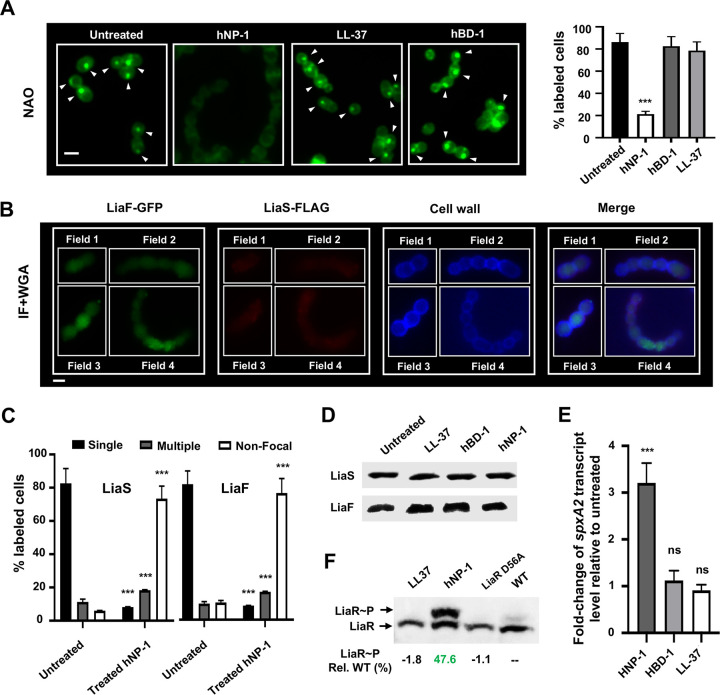
The human AMP hNP-1 activates the LiaFSR 3CS via disruption of LiaF and LiaS colocalization. (A) (Left panel) GAS cells were treated with hNP-1 (100 μg/ml), LL-37 (100 μg/ml), and hBD-1 (50 μg/ml) as described in Materials and Methods, and ExPortal (white arrowheads) was visualized by NAO staining. (Right panel) Quantification of labeled cells was performed using a minimum of 300 cells and three independent experiments (means ± SD). Scale bar, 1 μm. (B) GAS strains expressing LiaF-GFP and LiaS-FLAG were treated with hNP-1 (100 μg/ml), and the distribution was detected by IF microscopy as described in Materials and Methods. Cells were counterstained with WGA-Alexa Fluor 350 conjugate (blue). Images are merged as indicated at the top of the panel. Four independent fields are shown. Scale bar, 1 μm. (C) The distribution of LiaS and LiaF was quantitated by IF microscopy and shown as means ± SD of results from a minimum of 300 stained cells from three independent experiments. Quantitative data from untreated cells were obtained from the experiments represented in Fig. 2A and B. (D) The effect of human AMPs on LiaF and LiaS cellular protein levels. Following exposure to human AMPs as described for panels A to C, LiaF-GFP and LiaS-FLAG were monitored by Western blotting using an anti-GFP antibody and anti-FLAG antibody. (E) The activation of LiaFSR 3CS in the presence of hNP-1, hBD-1, and LL-37 was determined by qRT-PCR analysis for *spxA2* as described in Materials and Methods. Data are shown as means ± SD of results from three independent experiments. (F) Determination of LiaR phosphorylation (LiaR∼P) levels using Phos-tag gel. LiaR∼P levels (percent relative to WT, untreated) are indicated below the individual lanes. *P* values were determined by Student's *t* test. ***, *P < *0.001 relative to untreated. ns, not significant.

Previously, it was shown that human AMPs affect ExPortal integrity (11). Thus, we sought to examine the effect of the human AMPs hNP-1 (alpha-defensin), hBD-1 (beta-defensin), and LL-37 (cathelicidin) on LiaFSR activation. We first evaluated the effect of the AMPs on GAS growth *in vitro*. Only the alpha-defensin hNP-1 demonstrated growth inhibition (Fig. S6). NAO staining of cells following exposure to individual AMPs at the maximal amount that did not impact growth (Fig. S6) revealed ExPortal disruption by hNP-1 but not by hBD-1 or LL-37 (Fig. 3A) and was similar to that observed for antimicrobials (Fig. 2A and B). *In vitro* exposure to hNP-1 resulted in the redistribution of LiaF and LiaS, resulting in multiple foci or a diffuse pattern (Fig. 3B and C). As seen following antimicrobial treatment, exposure to human AMPs did not alter LiaF or LiaS protein levels (Fig. 3D). LiaFSR activation, as assessed by *spxA2* transcript level, was observed only following the addition of hNP-1 (Fig. 3E). No significant changes in *spxA2* transcript levels were observed in the presence of hBD-1 or LL-37 (Fig. 3E). Importantly, the concentration at which hNP-1 inhibition was observed (>50 μg/ml) was similar to that expected in purulent fluid (28). Finally, we assessed the LiaR phosphorylation level following hNP-1 exposure and observed significantly increased phosphorylation compared to controls (untreated and phosphorylation-deficient LiaR mutant) (Fig. 3F). Thus, our data confirm ExPortal sensitivity to the human AMP hNP-1 and support the idea of LiaFSR activation as a result of ExPortal disruption and LiaF/LiaS redistribution in the cell membrane.

10.1128/mBio.01804-20.6 FIG S6 Effect of human AMPs on GAS cell growth. MGAS10870 was grown in rich medium (THY). Download FIG S6, DOCX file, 0.3 MB. Copyright © 2020 Lin et al. 2020 Lin et al. This content is distributed under the terms of the Creative Commons Attribution 4.0 International license.

### Elimination of membrane cardiolipin results in the loss of LiaS/ExPortal colocalization.

**FIG 4:**
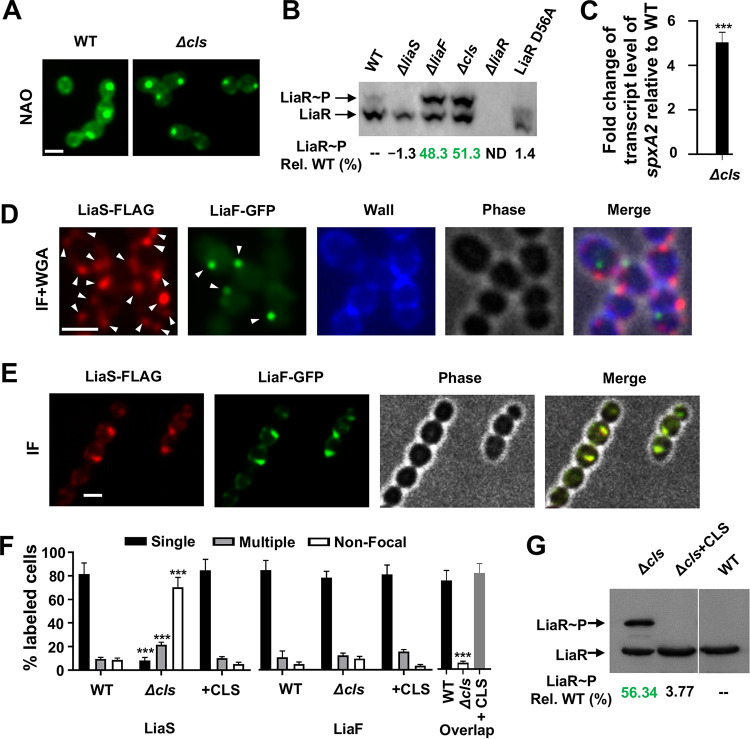
Absence of cardiolipin synthase alters the LiaS localization pattern. (A) NAO staining of GAS WT and Δ*cls* mutant. Scale bar, 1 μm. (B) Determination of LiaR phosphorylation (LiaR∼P) levels using Phos-tag gel. LiaR∼P levels (% relative to WT) are indicated below the individual lanes. (C) Activation of LiaFSR in the Δ*cls* mutant assessed by *spxA2* transcript level using qRT-PCR as described in Materials and Methods. (D) GAS cells (Δ*cls*) with LiaS-FLAG and LiaF-GFP were immunostained as described in Materials and Methods. Cell walls were visualized using WGA-Alexa Fluor 350. (E) GAS cells (Δ*cls*) carrying pLZ12Km2::CLS with LiaS-FLAG and LiaF-GFP were immunostained as described in Materials and Methods. (F) Quantification of LiaF-FLAG and LiaS-GFP focalization using immunostaining as described for panels D and E. Quantitative data represent means and SD of results from at least three independent experiments and examination of a minimum of 300 stained cells. WT quantitative data were obtained from the experiments represented in Fig. 1C and D. (G) Determination of LiaR phosphorylation (LiaR∼P) levels in the GAS WT strain, the Δ*cls* mutant strain, and a Δ*cls* mutant strain carrying pLZ12Km2::CLS using Phos-tag gel. LiaR∼P levels (percent relative to WT) are indicated below the individual lanes. *P* values were determined by Student's *t* test. ***, *P < *0.001 relative to WT.

Cardiolipin (CL) is an anionic phospholipid known to contribute to membrane protein stability and localization (29). Phosphatidylglycerol (PG) may be converted to CL by cardiolipin synthase (CLS), of which GAS genomes possess a single copy (30). Deletion of *cls* (Δ*cls*) in GAS results in complete loss of membrane CL, but the ExPortal remains intact, indicating that additional factors contribute to maintenance of the membrane microdomain (30). We hypothesized that LiaF and/or LiaS association with the ExPortal was CL dependent. We tested our hypothesis by constructing an isogenic mutant lacking CLS (Δ*cls*, Table S1) that was subsequently used to introduce GFP- and FLAG-tagged LiaF and LiaS proteins, respectively, as shown in Fig. 1. The mutant lacking CLS (Δ*cls*) showed a focal staining pattern similar to that seen with the parental strain when stained with NAO, indicating an intact ExPortal (Fig. 4A). The Δ*cls* strain contained high levels of phosphorylated LiaR consistent with LiaFSR activation (Fig. 4B). In contrast, a strain lacking the HK (Δ*liaS*) or mutated at the essential aspartate (D) residue in LiaR (LiaR-D56A) showed WT levels of LiaR phosphorylation (Fig. 4B). Similarly to the dissociation of LiaF and LiaS and resultant LiaFSR activation observed following antimicrobial or AMP exposure (Fig. 2C; see also Fig. 3E), we observed significantly increased *spxA2* transcript levels in the Δ*cls* mutant compared to the WT parental strain (Fig. 4C). Immunofluorescence microscopy showed that the Δ*cls* strain contained single LiaF foci but had multiple points for LiaS (Fig. 4D) that did not overlap (Fig. 4F). LiaF also demonstrated colocalization with HtrA in the Δ*cls* strain, verifying an association with the ExPortal (Fig. S7). Consistent with a cardiolipin-specific effect, complementation of the Δ*cls* mutant in *trans* (Table S1) showed a restoration of single LiaF and LiaS foci with colocalization by IF microscopy (Fig. 4E and F). In addition, we observed significantly decreased LiaR phosphorylation upon complementation of the Δ*cls* mutant that was similar to that seen with the WT (Fig. 4G). The data indicate that cardiolipin is critical for LiaS association with the GAS ExPortal, thereby maintaining LiaFSR activation in the basal state.

10.1128/mBio.01804-20.7 FIG S7 LiaF colocalization with HtrA in cardiolipin-deficient strain. Download FIG S7, DOCX file, 1.1 MB. Copyright © 2020 Lin et al. 2020 Lin et al. This content is distributed under the terms of the Creative Commons Attribution 4.0 International license.

### Mutants lacking LiaF exhibit altered ExPortal integrity.

**FIG 5:**
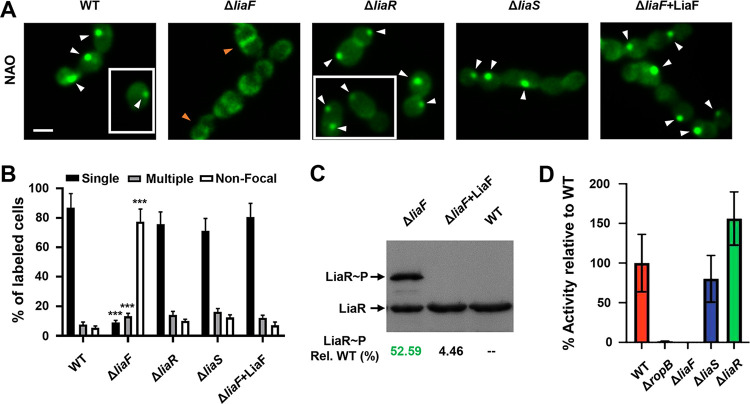
Absence of LiaF results in disruption of the GAS ExPortal. (A) NAO staining of GAS WT or isogenic deletion mutant cells as described in Materials and Methods. Arrowheads indicate focal staining consistent with ExPortal (white arrowheads) or nonfocal, septal staining (orange arrowheads). White boxes indicate additional fields of view from the same experiment. (B) The distribution of ExPortal was manually quantified by fluorescence microscopy as described for panel A. Data are shown as means ± SD of results from a minimum of 300 stained cells from three independent experiments. (C) Determination of LiaR phosphorylation (LiaR∼P) levels in the GAS WT, the Δ*liaF* mutant, and a Δ*liaF* mutant strain carrying pLZ12Km2::LiaF using Phos-tag gel. LiaR∼P levels (% relative to WT) are indicated below the individual lanes. (D) SpeB activity determined using FITC-casein assay as described in Materials and Methods. Shown is SpeB activity from culture supernatants relative to the parental, WT strain from three biological replicates (means and SD). The isogenic Δ*ropB* deletion strain (regulator of SpeB) was included as a negative control. *P* values were determined by Student's *t* test. ***, *P < *0.001 relative to WT.

Membrane microdomain stability may also be driven through specific protein-protein interaction (3). Thus, we next hypothesized that LiaF and/or LiaS may contribute to the maintenance of the GAS ExPortal. We generated isogenic deletion mutants lacking LiaF (Δ*liaF*) or LiaS (Δ*liaS*) in the parental strain and performed NAO staining to visualize the ExPortal. The mutant lacking LiaS (Δ*liaS*) showed a focal staining pattern similar to the parental strain pattern (Fig. 5A and B). However, in contrast to that observed for the WT and Δ*liaS* strains, the Δ*liaF* strain displayed primarily septal and circumferential cell staining that was restored upon complementation in *trans* (Fig. 5A and B). We also compared the NAO staining patterns of the Δ*liaF* and Δ*liaS* strains to that of a mutant lacking the response regulator (Δ*liaR*)—a cytoplasmic protein with no known membrane association—and observed a WT staining pattern in the Δ*liaR* strain (Fig. 5A and B). Complementation of the Δ*liaF* mutant also restored WT levels of LiaR phosphorylation (Fig. 5C). Consistent with disruption and the requirement for an intact ExPortal for SpeB (cysteine protease) secretion (10), we observed nearly absent SpeB activity in the Δ*liaF* mutant but not the Δ*liaS* mutant (Fig. 5D) similar to that seen with a mutant lacking the essential regulator for *speB* transcription the (Δ*ropB* mutant; Fig. 5D) (31). In total, the data derived from Δ*cls*, Δ*liaF*, and Δ*liaS* mutants suggest distinct mechanisms contributing to microdomain association in GAS.

## DISCUSSION

**FIG 6:**
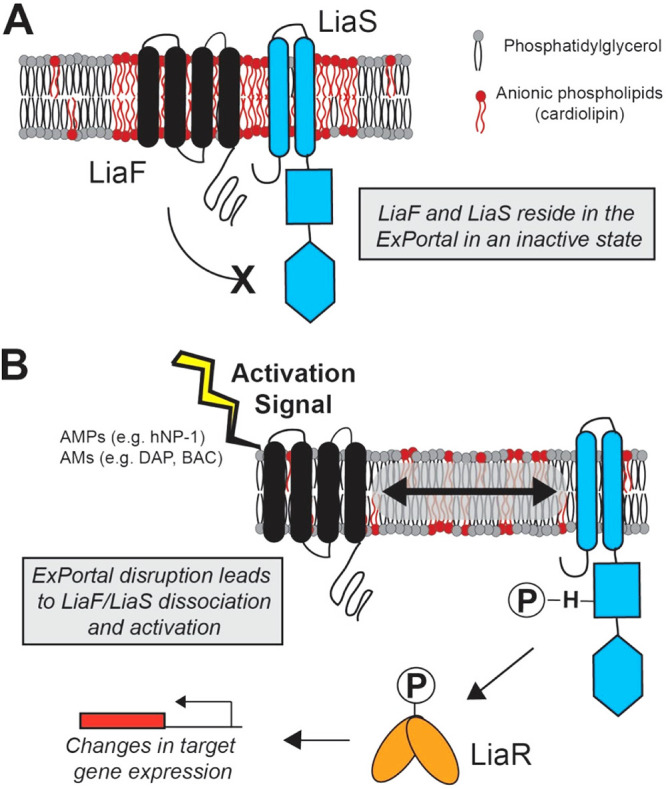
Model of ExPortal-LiaFSR codependence in sensing membrane stress in GAS. (A) In an unstressed state, LiaF and LiaS reside in association with the GAS ExPortal, defined by locally higher concentrations of anionic phospholipids, including cardiolipin. In this state, LiaF acts as an inhibitor of LiaS activity. (B) Antimicrobials (AMs; daptomycin [DAP] and bacitracin [BAC]) and antimicrobial peptides (AMPs; human neutrophil peptide 1 [hNP-1]) act as activation signals disrupting the GAS ExPortal, resulting in LiaF/LiaS dissociation. Upon dissociation, LiaS becomes activated and undergoes phosphorylation and phosphorylates LiaR, leading to transcriptional changes in target genes (e.g., *spxA2*). LiaS most likely exists as a dimer but for simplicity is shown as a monomer. The LiaF/LiaS ratio in GAS is unknown but, based on studies in *Bacillus* (38), may be present in relative excess. For simplicity, the LiaF/LiaS ratio is represented as 1:1.

Sensing of environment is critical to bacterial pathogenesis. Unique among TCSs are intramembrane-sensing histidine kinases as they lack external sensing domains and instead rely upon changes within the cell membrane and accessory proteins to trigger the cascade of events leading to a transcriptional response (16). LiaFSR, found only in Gram-positive bacteria, is one such system and has garnered much interest due to its role in the development of antibiotic resistance (17, 32–34). The current investigation provides evidence that the histidine kinase, LiaS, and the accessory protein, LiaF, of the human pathogen Streptococcus pyogenes reside within a discrete membrane microdomain, the ExPortal. In response to antimicrobial- or AMP-induced ExPortal stress, LiaFSR acts as a critical signal transducer to coordinate the bacterial response (Fig. 6). Surprisingly, the data support the essentiality of LiaF for membrane microdomain integrity in GAS. Combined with data indicating that LiaF (YvqF/VraT) and LiaS (VraS) homologs colocalize with membrane microdomains in S. aureus (3), the current report provides a model applicable to many Gram-positive pathogens.

In addition to the association with the GAS ExPortal, our data suggest distinct mechanisms by which LiaF and LiaS localize within the cell membrane and potential roles in microdomain formation. Two primary mechanisms are attributed to protein focalization and membrane microdomain formation: differences in lipid content and protein-protein interaction (35). Constituent lipids of bacterial microdomains include carotenoids and cardiolipin, which are critical for microdomain formation in Staphylococcus aureus (3) and in Escherichia coli and *Bacillus* (36, 37), respectively. Our data are consistent with this concept in that the LiaS protein mislocalizes in a strain lacking the only known GAS cardiolipin synthase, suggesting a critical role of cardiolipin in LiaS association with the ExPortal. It is now recognized that bacterial flotillins are critical for protein aggregation at membrane microdomains promoting protein-protein interactions (3). That a GAS strain lacking LiaF exhibits complete ExPortal disruption suggests a possible role of LiaF in maintaining microdomain assembly. In *Bacillus*, LiaF is in relative excess compared to LiaS (estimated 14:1 ratio) (38). Thus, if also present in relatively large amounts in the GAS ExPortal, LiaF may promote the membrane lipid and protein aggregation essential for microdomain formation. Alternatively, given that the absence of LiaF results in constitutive activation of LiaS and LiaR, changes in gene transcription resulting in altered cell membrane lipid composition may affect microdomain assembly. Further work is needed to determine the role of LiaFSR in microdomain formation in GAS and other Gram-positive bacteria.

The finding that inhibitors of cell wall synthesis such as vancomycin disrupt the GAS ExPortal is of particular interest in the climate of antimicrobial resistance. Disassembly of the GAS ExPortal is likely related to the key association of ExPortal integrity and an intact peptidoglycan layer (7). The GAS organism remains quite susceptible to beta-lactam antibiotics despite recent reports of penicillin-binding protein mutations resulting in modestly increased MICs (39). However, studies in methicillin-resistant Staphylococcus aureus (MRSA) showed that targeting the FMM leads to a resensitization to beta-lactams, primarily through disruption of PBP2a oligomerization (3, 40). This so-called seesaw effect is exploited to treat recalcitrant MRSA infections (41). Together, these findings further imply a common theme among Gram-positive pathogens that can be exploited to combat resistance.

Our data indicate a refined response by GAS to human AMPs. GAS is a human-specific pathogen, and centuries of coevolution have resulted in specificity of GAS proteins for human molecules. Previously, it was demonstrated that the human cathelicidin LL-37 induces CovRS-mediated virulence gene expression (42). Subsequently, the same group showed that a specific fragment of LL-37 interacts with the extracellular domain of CovS, resulting in altered virulence gene expression (15). Our data indicate that, consistent with activation of intramembrane-sensing TCSs (16), LiaFSR becomes activated upon membrane perturbation (assessed by ExPortal/FMM integrity) by a specific human AMP, in this case hNP-1. Given that the primary reservoir of hNP-1 in the human host is the neutrophil (43), the effect of hNP-1 on the ExPortal and LiaFSR activation (relative to that seen with hBD-1 or LL-37) highlights the importance of the GAS-neutrophil interaction in GAS infections.

In summary, we describe a heretofore-unrecognized mechanism by which GAS responds to the human host by linking specific membrane perturbations (FMM) with TCS activation (LiaFSR). The critical role of the membrane microdomains in bacterial physiology and the high degree of LiaFSR conservation suggest a common theme among Gram-positive bacterial pathogens.

## MATERIALS AND METHODS

### Bacterial strains and growth conditions.

MGAS10870, a serotype M3 GAS invasive strain isolated in 2002 from an individual with a soft tissue infection in Ontario, Canada (44), was used throughout the study. All intermediate and final strains synthesized are listed in Table S1 in the supplemental material. All GAS strains were grown at 37°C in Todd-Hewitt broth supplemented with 0.2% (wt/vol) yeast extract (THY; Difco Laboratories, Detroit, MI), on THY agar, or on BD Trypticase soy agar II with 5% sheep blood (sheep blood agar [SBA]; Becton Dickinson, Franklin Lakes, NJ). All cloning was performed in Escherichia coli strain NEB5alpha (New England Biolabs, Ipswich, MA) in Luria-Bertani (LB) broth or on LB plates (0.8% agar) (Difco Laboratories). Growth of all strains took place at 37°C with 5% CO_2_. In selected experiments, media were supplemented with antimicrobial peptides, including daptomycin (Sigma-Aldrich, St. Louis, MO), polymyxin B (Sigma-Aldrich), colistin (Sigma-Aldrich), nisin (Sigma-Aldrich), vancomycin (Sigma-Aldrich), bacitracin (Sigma-Aldrich), tetracycline (Sigma-AnaSpec), human neutrophil peptide‐1 (hNP‐1; AnaSpec, Freemont, CA), human β‐defensin 1 (hBD‐1; Phoenix Pharmaceuticals, Burlingame, CA), and human cathelicidin LL‐37 (LL‐37; AnaSpec) at the concentrations indicated in the text.

### Generation of isogenic mutants in MGAS10870.

The plasmids and primers used for isogenic mutant generation are listed in Tables S2 and S3, respectively. Gene fusions to generate GFP- and FLAG-labeled proteins and in-frame gene deletions were constructed using overlap sewing PCR (45). Strains expressing markerless superfolded green fluorescent protein (sfGFP) or 3xFLAG (FLAG) (Table S1) were generated using the allelic exchange plasmid pJL1055 as described previously (46). The in-frame deletions of *liaF*, *liaS*, *liaR*, *cls*, and *ropB* were made by replacement with either a kanamycin resistance gene (*aph* or *ropB*; Table S1) or a spectinomycin resistance gene (*aad9*) in the MGAS10870 parental strain. All isogenic mutant strains underwent whole-genome sequencing for confirmation and lacked spurious mutations.

10.1128/mBio.01804-20.9 TABLE S2 Plasmids used in this study. Download Table S2, DOCX file, 0.02 MB. Copyright © 2020 Lin et al. 2020 Lin et al. This content is distributed under the terms of the Creative Commons Attribution 4.0 International license.

10.1128/mBio.01804-20.10 TABLE S3 Oligonucleotides used in this study. Download Table S3, DOCX file, 0.02 MB. Copyright © 2020 Lin et al. 2020 Lin et al. This content is distributed under the terms of the Creative Commons Attribution 4.0 International license.

### Challenge with antimicrobials and AMPs.

Unless otherwise indicated, bacteria were cultured overnight in liquid THY medium, diluted 1:100 in fresh, prewarmed THY medium, and then cultured to the early logarithmic phase of growth (optical density at 600 nm [OD_600_] of 0.4). The antimicrobial or AMP was then added to the final concentration indicated, and incubation continued for an additional hour. Untreated control cultures were grown in parallel. At selected time points following challenge with antimicrobials or AMPs, aliquots were removed from cultures. LiaFSR 3CS activity was monitored by the use of quantitative real-time PCR (qRT-PCR) or Pho-tag gels as described below. Protein distribution and ExPortal were visualized by immunofluorescence or NAO staining as described below. Viability was assessed by determination of CFU following brief vortex mixing for 5 min to disrupt streptococcal chains, serial dilution in phosphate-buffered saline (PBS), and plating on SBA. Data reported represent means and standard errors of the means derived from a minimum of three independent experiments.

### Dansyl-polymyxin B synthesis.

Dansyl-polymyxin B (dPM) was synthesized as described previously (47) with minor modifications. Briefly, polymyxin B sulfate (Sigma-Aldrich) was dissolved in 0.1 M NaHCO_3_ to reach a final concentration of 26 mM. Dansyl chloride (Acros Organics, Thermo Fisher Scientific, Waltham, MA) was dissolved in acetone to reach a final concentration of 46 mM. Polymyxin B was mixed with dansyl chloride at a molecular ratio of 3:2, and the mixture was placed in the dark for 120 min at 37°C. After incubation, the dPM and the unreacted dansyl chloride were separated by passage through a Sephadex G-25 column (Sigma-Aldrich). The fractions containing the dPM were extracted into n-butanol. The pure dPM was dissolved in the buffer of 5 mM HEPES (pH 7.0) and stored in aliquots at −20°C. The concentration of the dPM was determined by dinitrophenylation assay (48).

### Cellular staining and fluorescence microscopy.

The location and integrity of a membrane microdomain enriched in anionic phospholipids were assessed by staining with 10‐nonyl acridine orange (NAO; Invitrogen) (30) or dPM (11). Briefly, strains were grown to an OD_600_ of 0.4 and NAO or dPM was added to reach a final concentration of 1 μM or 10 μM, respectively. Cells were incubated an additional 30 min at 37`συπ>ò/συπ>C and were harvested by centrifugation. After three washes with PBS, cells were attached to glass slides pretreated with poly-l-lysine. Samples were examined using a Keyence model BZ-X700 all-in-one fluorescence microscope and images captured using BZ-X Analyzer software (Keyence, Itasca, IL). Where indicated, simultaneous treatment with two reagents was conducted in order to assess colocalization of staining. In such experiments, GAS cell walls were visualized by staining with wheat germ agglutinin (WGA) Alexa Fluor 350 conjugate (5 μg/ml; Invitrogen, Thermo Fisher). Focal localization in images of cells was quantified by scoring staining at a unique (single) focus, at multiple foci, or nonspecifically (the staining was of homogeneous intensity around the cellular circumference). Data presented for each condition represent means and standard errors of the means of results derived from at least three independent experiments and examination of a minimum of 300 stained cells as indicated in the text.

### Immunofluorescent microscopy.

Aliquots from untreated or antimicrobial/AMP-treated cultures (see above) were collected as indicated and centrifuged to pellet cells. For fixation, cells were resuspended in 100 mM phosphate buffer (pH 7.4) containing 4% paraformaldehyde (Avantor, VWR, Radnor, PA) and 0.5% glutaraldehyde (Fisher Scientific) and incubated for 1 h at 4°C. After fixing, cells were washed three times with 0.1 M phosphate buffer (pH 7.4) and attached to poly-l-lysine-coated glass slides. To increase the permeabilization of cells, the fixed cells were treated with ice-cold methanol following the treatment of PlyC lysin (a generous gift from Daniel C. Nelson, University of Maryland, USA) (49). Antisera used included a polyclonal rabbit anti‐GFP antiserum (Abcam, Cambridge, MA) used at a dilution of 1:200 that was detected using Alexa Fluor 488‐labeled goat anti‐rabbit IgG (Abcam) at 1:500 and a polyclonal mouse anti‐FLAG antiserum (Invitrogen, Thermo Fisher) used at a dilution of 1:200 that was detected using Alexa Fluor 647‐labeled goat anti‐mouse IgG (Abcam) at 1:500. Slides were mounted in an antifade medium (Vector Laboratories, Burlingame, CA) and images captured and staining patterns quantified as described for NAO staining above.

### Immunogold labeling and transmission electron microscopy.

Cells were prepared and fixed as described above. Samples were then dehydrated and infiltrated with LR White resin (Electron Microscopy Sciences, Hatfield, PA) as follows: 50% ethanol for 15 min, 70% ethanol for 15 min, 80% ethanol for 10 min, LR White resin/ethanol (2:1 LR White resin to 70% [vol/vol] ethanol) for 60 min, 100% LR White resin for 60 min, 100% LR White resin overnight, 100% LR White resin for 30 min, and 100% LR White resin for 30 min. Samples were then embedded in fresh LR White resin and polymerized at 55°C for 72 h. Samples were sectioned with a Leica Ultracut UCT ultramicrotome in 70-to-80-nm sections and immunogold labeled with a polyclonal rabbit anti‐GFP antiserum (Abcam) or a polyclonal mouse anti‐FLAG antiserum (Invitrogen, Thermo Fisher) followed by 12-nm-diameter colloidal gold-conjugated goat anti-rabbit IgG (Abcam) or 18-nm-diameter colloidal gold-conjugated donkey anti-mouse IgG (Abcam). Sections were stained with 1% uranyl acetate and 0.01% lead citrate for 10 and 5 min, respectively, and viewed on a JEOL JEM 1400 120-kV transmission electron microscope. Double immunolabeling was performed using the two primary antibodies in tandem followed by 18-nm-diameter colloidal gold-conjugated donkey anti-mouse IgG and 12-nm-diameter colloidal gold-conjugated goat anti-rabbit IgG. All labeling experiments were processed in parallel omitting one primary antibody. These controls were consistently negative for the corresponding secondary gold-conjugated antibody (data not shown).

### GAS membrane ExPortal isolation.

GAS cultures were prepared as described above. Pellets were resuspended in PBS buffer supplemented with 1 mM phenylmethylsulfonyl fluoride (PMSF), 20 μg/ml DNase I (Sigma), and 5 μg/ml PlyC lysin and incubated for 30 min at 25°C. Cell suspensions were disrupted using a French press. Unbroken cells and debris were removed by centrifugation (10 min, 16,000 × *g*, 4°C). Membrane fractions were collected by ultracentrifugation (1 h, 100,000 × *g*). For ExPortal isolation, the membrane fractions were processed using a CelLytic MEM protein extraction kit (Sigma). The detergent-resistant fraction (DRF) and detergent-sensitive fraction (DSF) were separated according to the manufacturer’s protocol.

### Extraction of RNA for quantitative real-time PCR.

Samples from GAS cultures grown for NAO staining or immunofluorescence were harvested by centrifugation, and RNA was isolated and purified with a RNeasy minikit (Qiagen, Germantown, MD) according to manufacturer protocol with modifications for Gram-positive bacteria. The quantity and quality of RNA were determined using a NanoDrop spectrophotometer (Thermo Fisher) and an Agilent 4200 TapeStation system (Agilent Technologies, Santa Clara, CA). TaqMan (Applied Biosystems, Thermo Fisher) quantitative real-time PCR (qRT-PCR) analysis of cDNA produced using SuperScript III reverse transcriptase (Invitrogen, Thermo Fisher) was performed with an CFX96 Touch real-time PCR detection system (Bio-Rad Laboratories, Hercules, CA) according to the manufacturer’s instructions, with 50 ng total RNA and a 10 nM concentration of each of the primers (forward and reverse) in a 20-μl volume. Real-time PCR primers and probes used in the analyses are listed in Table S3. Transcript levels of genes were calculated by relative quantification using the threshold cycle (ΔΔ*C_T_*) method as described previously (50), with a *tufA* internal reference gene used as the normalizing gene (51). All reactions were performed in technical triplicate using RNA purified from at least three biological replicates.

### SpeB protease activity assay.

SpeB protease activity was measured using fluorescein isothiocyanate (FITC)-labeled casein (G-Biosciences, St. Louis, MO) as a protease substrate. Supernatants of GAS strains grown overnight (∼16 h) in THY broth were obtained by centrifugation (4,000 × *g*, 4°C, 10 min), diluted in the same volume of freshly prepared activation buffer (1 mM EDTA, 20 mM dithiothreitol [DTT], 0.1 M sodium acetate, pH 5.0), and incubated for 30 min at 40°C. Subsequent addition of the same volume of activation buffer containing 0.02 μg/μl FITC-labeled casein was followed by further incubation for 30 min at 40°C. Proteolytic activity of SpeB against FITC-labeled casein was measured by fluorescence (485-nm-wavelength excitation, 535-nm-wavelength emission). Activity data shown represent means and standard errors of the means derived from triplicate determinations of samples and are representative of results from at least three independent experiments.

### Complementation of *cls* and *liaF* deletion mutants.

The *cls* and *liaF* genes, including their promoters, were PCR amplified (Table S3) from MGAS10870, digested with BamHI and with PstI, and ligated into the expression plasmid pLZ12Km2, resulting in pLZ12Km2::CLS or pLZ12Km2::LiaF (Table S2). For complementation studies, the mutant strains were transformed with plasmid pLZ12Km2::CLS or pLZ12Km2::LiaF, which carried an open reading frame (ORF) of the *cls* gene or *liaF* gene, respectively, under the control of its native promoter. The positive mutant colonies were selected on THY plates containing 150 μg ml^−1^ kanamycin. The identity of the clones was confirmed by PCR and sequencing.

### Detection of LiaR phosphorylation levels *in vivo*.

LiaR phosphorylation levels were determined using Phos-Tag. GAS lysates were prepared using a FastPrep-24 5G homogenizer (MP Biomedicals) and separated on 12.5% SuperSeq Phos-tag gels (Wako, USA). Un/phosphorylated LiaR species were detected using a polyclonal anti-LiaR antibody (Covance) and a ChemiDoc MP imaging system (Bio-Rad). All phosphorylation experiments were performed at least twice on independent samples.

### Statistics.

All statistical analyses were performed with Prism 8 software (GraphPad Software, San Diego, CA). Differences between mean values (continuous variables) were determined using a two-tailed Student's *t* test. A *P* value of <0.05 was considered significant for all tests.
