# Supplementary material for: ExPortal and the LiaFSR Regulatory System Coordinate the Response to Cell Membrane Stress in Streptococcus pyogenes
Source: mBio. 2020 Sep 15;11(5):e01804-20. doi: 10.1128/mBio.01804-20 (PMC7492735; doi:10.1128/mBio.01804-20)
Supplement: FIG S1 [file mBio.01804-20-sf001.docx]

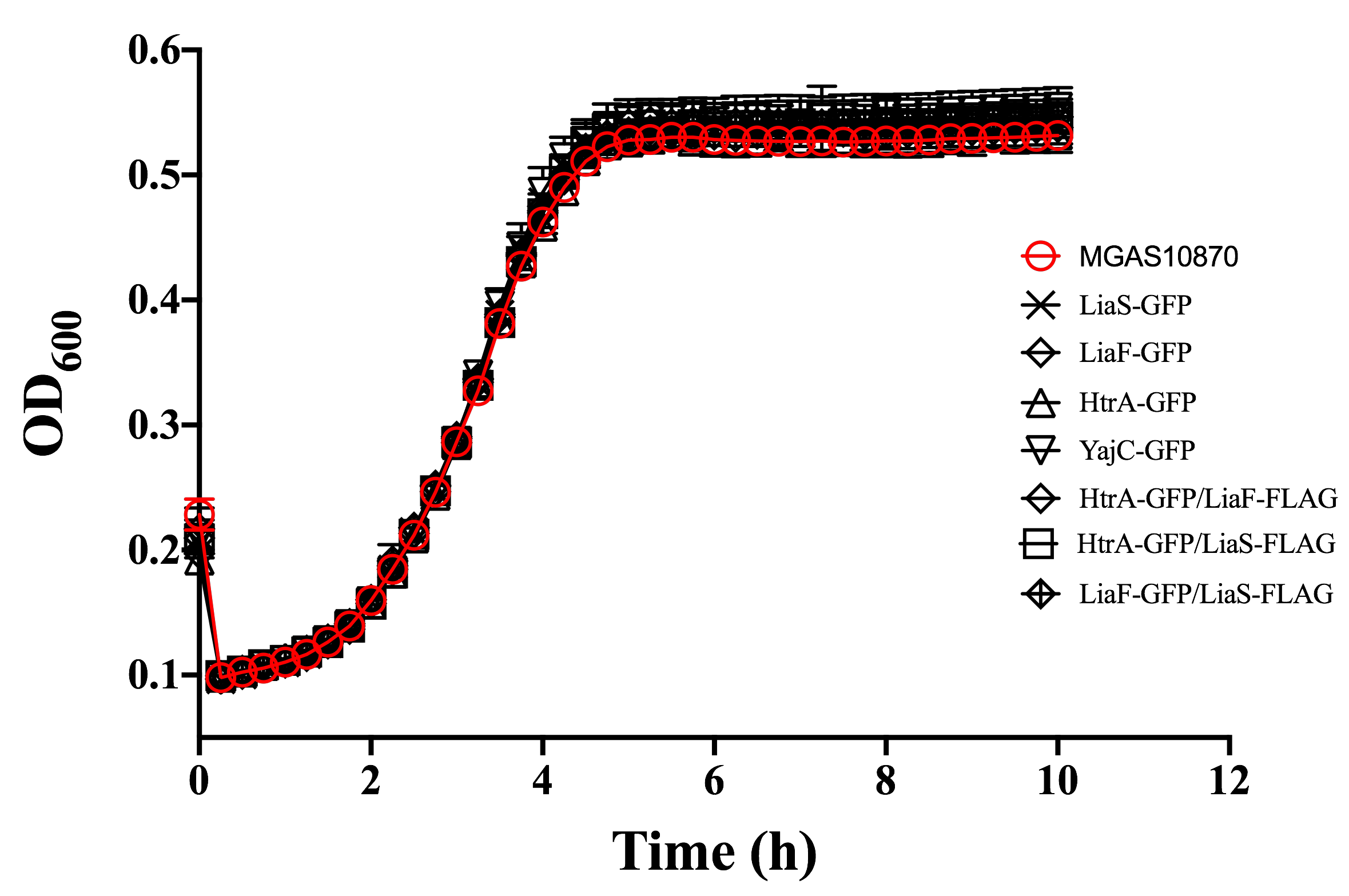


**Figure S1.** Growth curves of GFP-tagged proteins in GAS. LiaS-GFP, LiaF-GFP, HtrA-GFP, YajC-GFP, HtrA-GFP/LiaF-FLAG, HtrA-GFP/LiaS-FLAG, and LiaF-GFP/LiaS-FLAG expressing strains were grown in THY medium at 37°C.
