# Supplementary material for: ExPortal and the LiaFSR Regulatory System Coordinate the Response to Cell Membrane Stress in Streptococcus pyogenes
Source: mBio. 2020 Sep 15;11(5):e01804-20. doi: 10.1128/mBio.01804-20 (PMC7492735; doi:10.1128/mBio.01804-20)
Supplement: FIG S2 [file mBio.01804-20-sf002.docx]

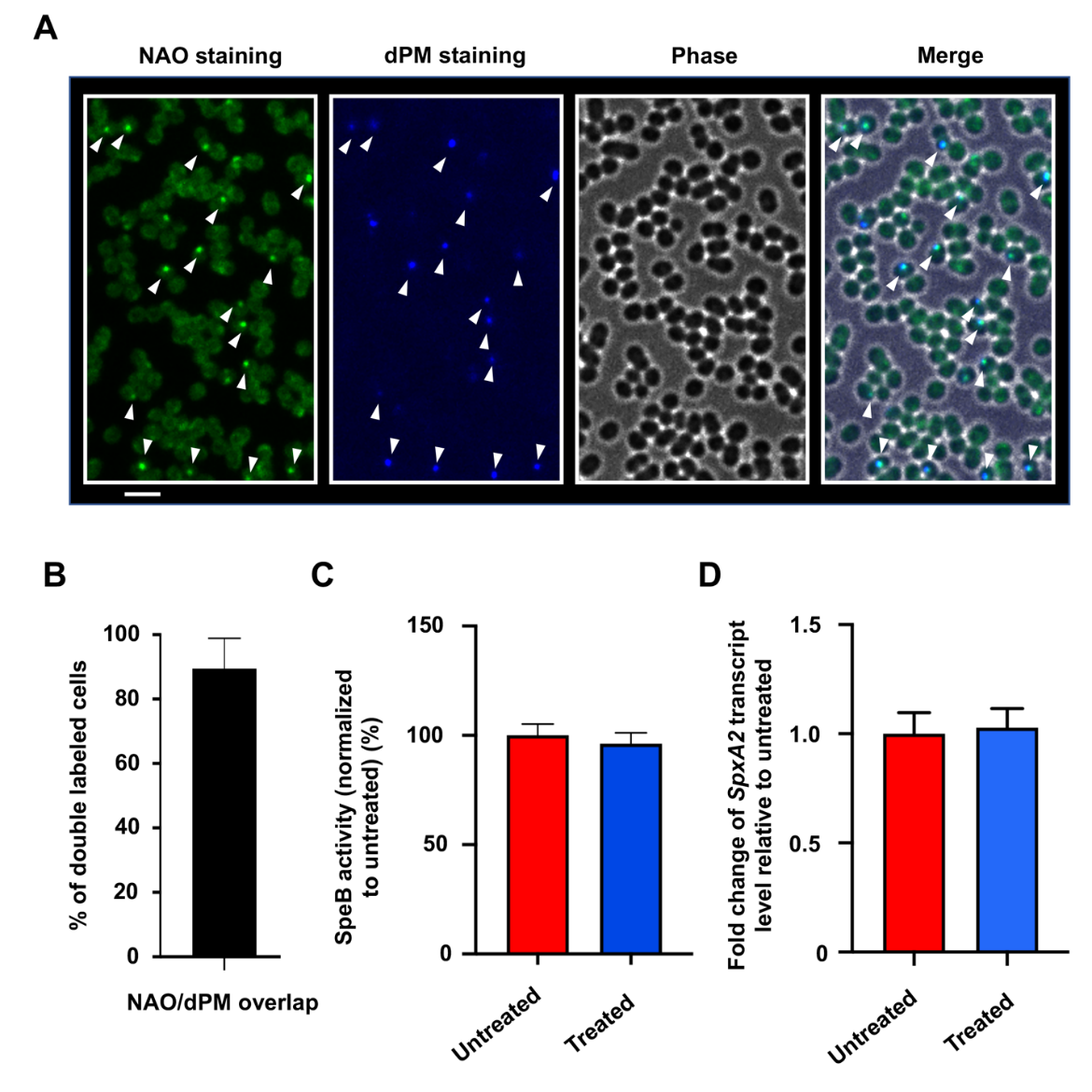


**Figure S2**. Dansyl-polymyxin B treatment does not alter membrane ExPortal profile. (**A**) Membrane ExPortal in GAS was stained with 1 µM of NAO (green), and a 10-µM concentration of dansyl-polymyxin B (dPM) (blue). Images (NAO staining and dPM staining) were merged with a phase-contrast image as indicated at the top. Arrowheads indicate NAO stained ExPortal and dPM stained ExPortal which are coincident. Scale bars, 2 µm. **(B)** The overlapping of NAO staining and dPM staining is quantified by fluorescence as described in (A). Data shown are mean ± SD of a minimum of 300 stained cells from three independent experiments. (**C**) SpeB protease activity using FITC-casein of GAS with or without dPM. (D) The gene-transcript levels of *spxA2* were quantitated (relative to an endogenous control *tufA*) in dPM treated sample and compared to the untreated sample.
