# Supplementary material for: ExPortal and the LiaFSR Regulatory System Coordinate the Response to Cell Membrane Stress in Streptococcus pyogenes
Source: mBio. 2020 Sep 15;11(5):e01804-20. doi: 10.1128/mBio.01804-20 (PMC7492735; doi:10.1128/mBio.01804-20)
Supplement: FIG S3 [file mBio.01804-20-sf003.docx]

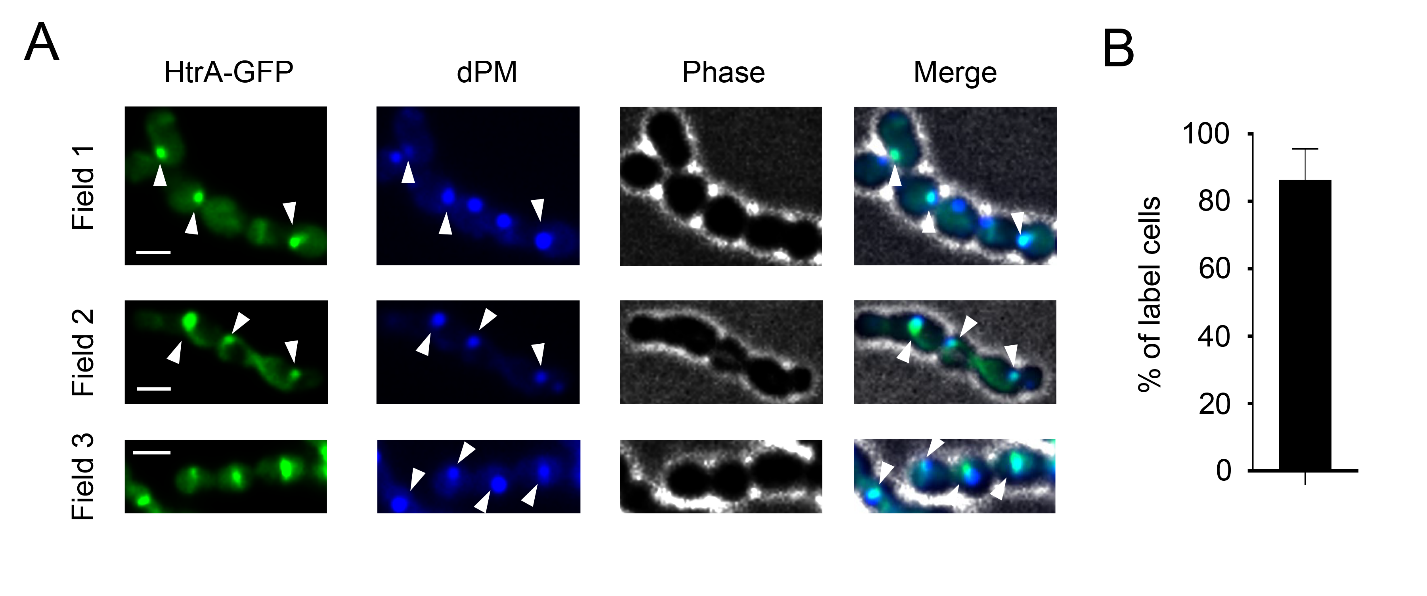


**Figure S3.** HtrA colocalizes with the ExPortal. (**A**) GAS strains expressing HtrA-GFP were stained with dansyl-polymyxin B (dPM) as described in Methods. Images were merged with a phase-contrast image as indicated above individual panels. Images collected from different fields are shown. Scale bar, 1 μm. (**B**) Colocalization of HtrA-GFP with ExPortal are quantified by fluorescence as described in (A). Data shown are mean ± SD of a minimum of 300 stained cells from three independent experiments.
