# Supplementary material for: ExPortal and the LiaFSR Regulatory System Coordinate the Response to Cell Membrane Stress in Streptococcus pyogenes
Source: mBio. 2020 Sep 15;11(5):e01804-20. doi: 10.1128/mBio.01804-20 (PMC7492735; doi:10.1128/mBio.01804-20)
Supplement: FIG S4 [file mBio.01804-20-sf004.docx]

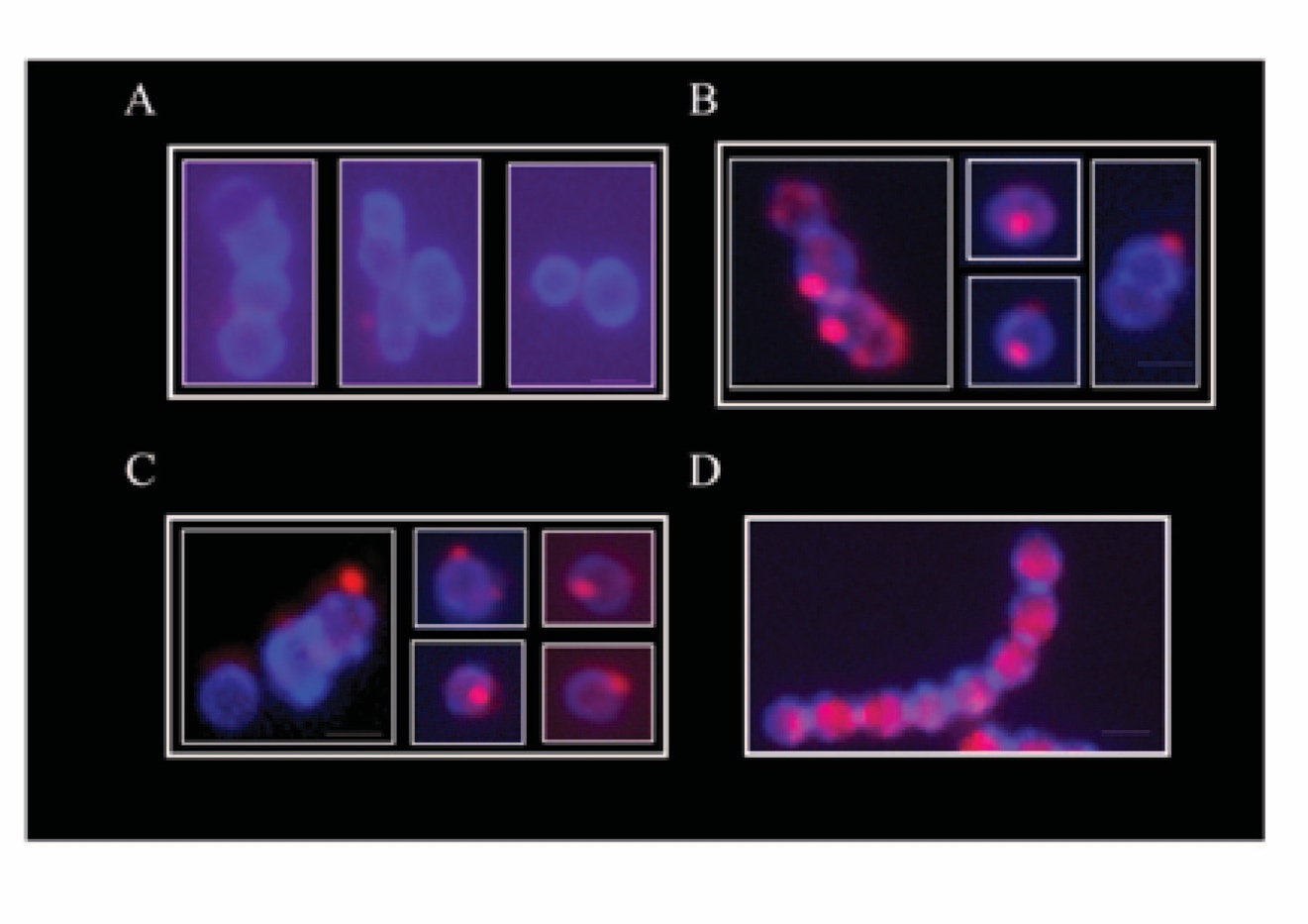


**Figure S4**. Focal localization of LiaS-FLAG and LiaF-FLAG. Localization of LiaS-FLAG (B), LiaF-FLAG (C), and YajC-FLAG (D) was monitored by IF microscopy. Cells were grown at 37°C until OD600 0.4 reach followed by immunofluorescence staining with a polyclonal mouse anti-FLAG antibody (Invitrogen) and an Alexa Fluor 647-labelled goat anti-mouse IgG (Abcam). The cells were counterstained with 5 μg/ml WGA Alexa Fluor 350 conjugate. MGAS10870 was used as negative control (A). For details see Materials and Methods.
