# Supplementary material for: ExPortal and the LiaFSR Regulatory System Coordinate the Response to Cell Membrane Stress in Streptococcus pyogenes
Source: mBio. 2020 Sep 15;11(5):e01804-20. doi: 10.1128/mBio.01804-20 (PMC7492735; doi:10.1128/mBio.01804-20)
Supplement: TABLE S1 [file mBio.01804-20-st001.docx]

|  | |  |  | |
| --- | --- | --- | --- | --- |
| Bacterial Strains | | Genotype/Description | Reference^a^ | |
| MGAS10870 | Wild-type, invasive, serotype M3 group A *Streptococcus* (GAS) | | | (43) |
| NEB 5-alpha | Competent *E. coli* strain used in cloning | | | New England BioLabs |
| LiaS-GFP | MGAS10870 with C-terminal LiaS sfGFP fusion | | | This study |
| LiaF-GFP | MGAS10870 with C-terminal LiaF sfGFP fusion | | | This study |
| HtrA-GFP | MGAS10870 with C-terminal HtrA sfGFP fusion | | | This study |
| YajC-GFP | MGAS10870 with C-terminal YajC sfGFP fusion | | | This study |
| LiaS-FLAG | MGAS10870 with C-terminal LiaS 3xFLAG fusion | | | This study |
| LiaF-FLAG | MGAS10870 with C-terminal LiaF 3xFLAG fusion | | | This study |
| HtrA-FLAG | MGAS10870 with C-terminal HtrA 3xFLAG fusion | | | This study |
| YajC-FLAG | MGAS10870 with C-terminal YajC 3xFLAG fusion | | | This study |
| HtrA-GFP/LiaS-FLAG | MGAS10870 with C-terminal HtrA sfGFP fusion and C-terminal LiaS 3xFLAG fusion | | | This study |
| HtrA-GFP/LiaF-FLAG | MGAS10870 with C-terminal HtrA sfGFP fusion and C-terminal LiaF 3xFLAG fusion | | | This study |
| LiaF-GFP/LiaS-FLAG | MGAS10870 with C-terminal LiaF sfGFP fusion and C-terminal LiaS 3xFLAG fusion | | | This study |
| Δ*cls* | MGAS10870 with in-frame deletion of *cls* using *aad9* (*Δcls:aad*9) | | | This study |
| Δ*cls*/LiaS-FLAG/LiaF-GFP | MGAS10870 Δcls strain with C-terminal LiaF sfGFP fusion and C-terminal LiaS 3xFLAG fusion | | | This study |
| Δ*cls*/LiaF-FLAG/HtrA-GFP | MGAS10870 Δcls strain with C-terminal HtrA sfGFP fusion and C-terminal LiaF 3xFLAG fusion | | | This study |
| Δ*liaF* | MGAS10870 with in-frame, unmarked deletion in *liaF* | | | This study |
| Δ*liaS* | MGAS10870 with in-frame deletion of *liaS* using *aad9* (Δ*liaS:aad9*) | | | This study |
| Δ*liaR* | MGAS10870 with in-frame deletion of *liaR* using *aad9* (Δ*liaR:aad9*) | | | This study |
| Δ*ropB* | MGAS10870 with in-frame deletion of *ropB* using *aph* (Δ*liaR*::*aph*) | | | This study |
| Δ*cls*/pLZ12Km2::CLS | MGAS10870 with in-frame deletion of *cls* using *aad9* (*Δcls:aad*9) carrying plasmid pLZ12Km2::CLS | | | This study |
| Δ*liaF*/pLZ12Km2::LiaF | MGAS10870 with in-frame, unmarked deletion in *liaF* carrying plasmid pLZ12Km2::LiaF | | | This study |
| LiaR-D56A | MGAS10870 with single amino acid change (aspartatic acid to alanine) at position 56 in LiaR | | | This study |

**Table S1**. Bacterial strains used in this study.

^a^ Numbers refer to citation in the main text.
