# Supplementary material for: ExPortal and the LiaFSR Regulatory System Coordinate the Response to Cell Membrane Stress in Streptococcus pyogenes
Source: mBio. 2020 Sep 15;11(5):e01804-20. doi: 10.1128/mBio.01804-20 (PMC7492735; doi:10.1128/mBio.01804-20)
Supplement: FIG S5 [file mBio.01804-20-sf005.docx]

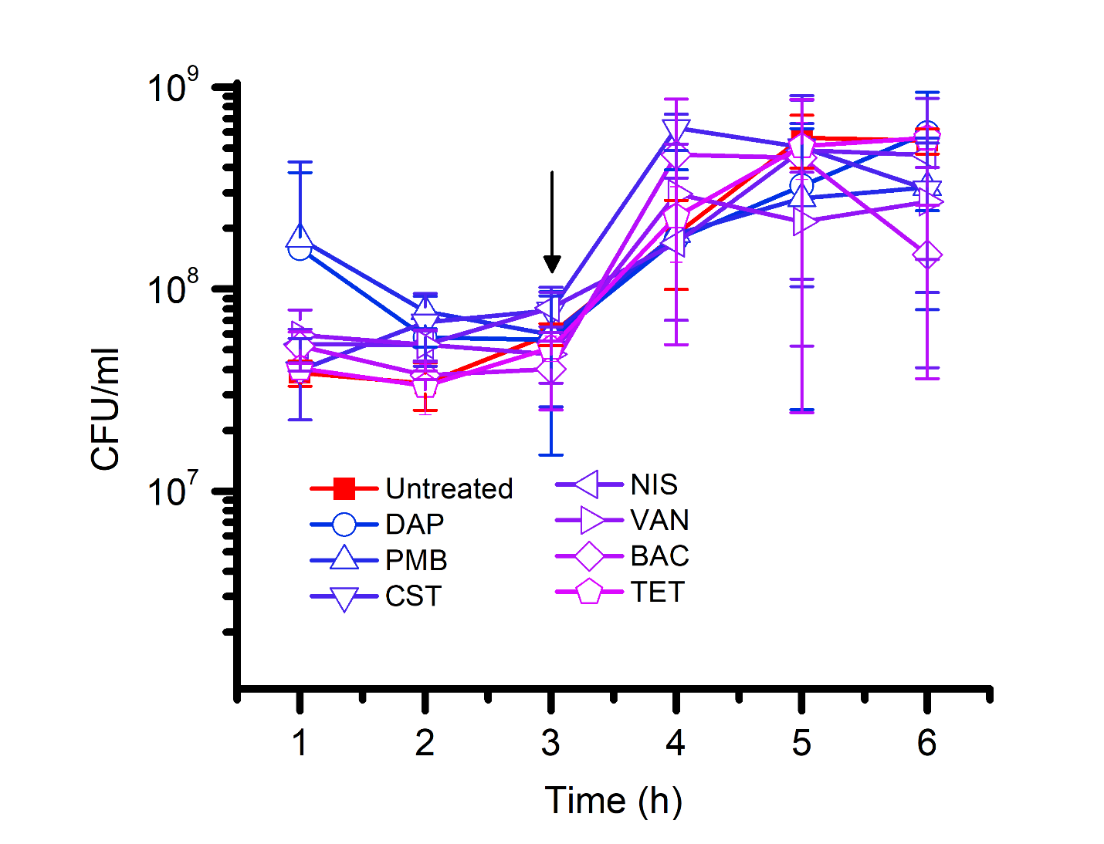


**Figure S5**. Colony forming unit (CFU) enumeration following growth in the presence of antimicrobials. Daptomycin (DAP; 0.4 μg/ml), polymyxin B (PMB; 50 μg/ml), cholistin (CST; 50 μg/ml), nisin (NIS; 4 μg/ml), vancomycin (VAN; 0.5 μg/ml), bacitracin (BAC; 1 μg/ml), tetracycline (TET; 1 μg/ml), or in media alone (untreated). Black arrow indicates the time point individual antibiotics were added.
