# Supplementary material for: ExPortal and the LiaFSR Regulatory System Coordinate the Response to Cell Membrane Stress in Streptococcus pyogenes
Source: mBio. 2020 Sep 15;11(5):e01804-20. doi: 10.1128/mBio.01804-20 (PMC7492735; doi:10.1128/mBio.01804-20)
Supplement: FIG S6 [file mBio.01804-20-sf006.docx]

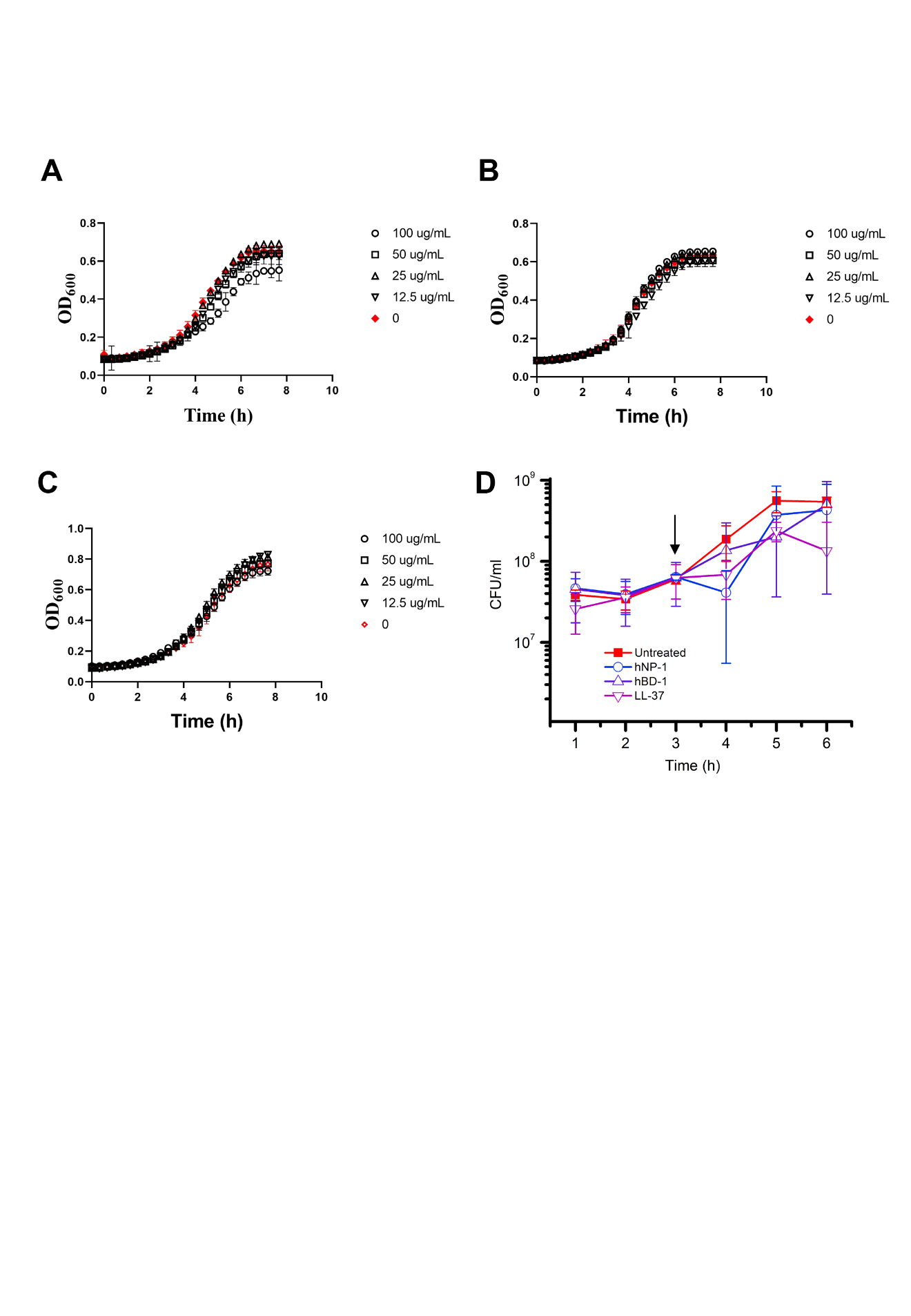


**Figure S6**. Effect of human AMPs on GAS cell growth. MGAS10870 was grown in rich medium (THY). Growth was measured in the presence of hNP-1 (A), hBD-1 (B), and LL-37 (C). (D) Colony forming unit (CFU) enumeration following growth in the presence of hNP-1 (100 μg/ml), hBD-1 (50 μg/ml), LL-37 (100 μg/ml) or without AMPs. AMPs were added at hour 3 timepoint for CFU enumeration.
