# Supplementary material for: ExPortal and the LiaFSR Regulatory System Coordinate the Response to Cell Membrane Stress in Streptococcus pyogenes
Source: mBio. 2020 Sep 15;11(5):e01804-20. doi: 10.1128/mBio.01804-20 (PMC7492735; doi:10.1128/mBio.01804-20)
Supplement: FIG S7 [file mBio.01804-20-sf007.docx]

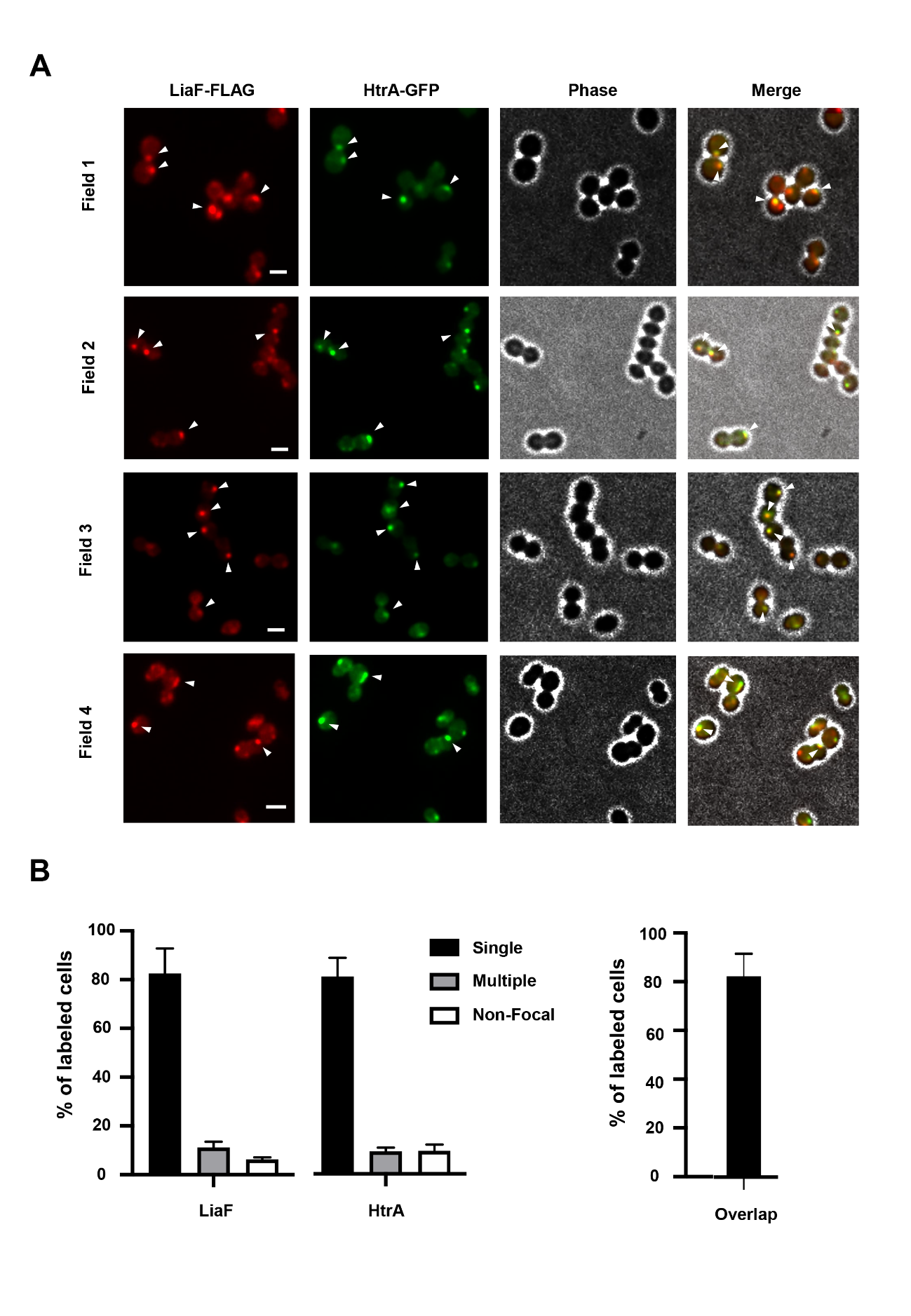


**Figure S7**. LiaF colocalization with HtrA in cardiolipin-deficient strain. (**A**) Cardiolipin-deficient (Δ*cls*) cells expressing LiaF-FLAG and HtrA-GFP were immunostained as described in Materials and Methods. Images were merged with a phase-contrast image as indicated above the individual panels. Cells from independent fields are indicated (field 1-4).  Arrowheads indicate a focal signal of LiaF-FLAG and HtrA-GFP. Scale bar, 1 um. (**B**) The colocalization of LiaF with HtrA was quantified by IF as described in (A). Data shown are mean ± SD of a minimum of 300 stained cells from three independent experiments.
