# Supplementary material for: ExPortal and the LiaFSR Regulatory System Coordinate the Response to Cell Membrane Stress in Streptococcus pyogenes
Source: mBio. 2020 Sep 15;11(5):e01804-20. doi: 10.1128/mBio.01804-20 (PMC7492735; doi:10.1128/mBio.01804-20)
Supplement: TABLE S2 [file mBio.01804-20-st002.docx]

**Table S2**. Plasmids used in this study.

| Plasmids | Description | Source/Reference | Use |
| --- | --- | --- | --- |
| pET28-sfGFP | sfGFP cloned between *Nde*I and *Xho*I | (23) | Cloning of sfGFP fusion proteins |
| pJL1055 | GAS/*E. coli* shuttle vector | (46) | Mutant generation in GAS |
| pCR-Blunt II-TOPO | Cloning vector | Invitrogen | Cloning |
| pJL1055::LiaF-sfGFP | LiaF-sfGFP cloned between *Bam*HI and *Xho*I | This study | LiaF-GFP mutant generation |
| pJL1055::LiaS-sfGFP | LiaS-sfGFP cloned between *Bam*HI and *Xho*I | This study | LiaS-GFP mutant generation |
| pJL1055::HtrA-sfGFP | HtrA-sfGFP cloned between *Bam*HI and *Xho*I | This study | HtrA-GFP mutant generation |
| pJL1055::YajC-sfGFP | YajC-sfGFP cloned between *Bam*HI and *Xho*I | This study | YajC-GFP mutant generation |
| pJL1055::LiaF-FLAG | LiaF-FLAG cloned between *Bam*HI and *Xho*I | This study | LiaF-FLAG mutant generation |
| pJL1055::LiaS-FLAG | LiaS-FLAG cloned between *Bam*HI and *Xho*I | This study | LiaS-FLAG mutant generation |
| pJL1055::HtrA-FLAG | HtrA-FLAG cloned between *Bam*HI and *Xho*I | This study | HtrA-FLAG mutant generation |
| pJL1055::YajC-FLAG | YajC-FLAG cloned between *Bam*HI and *Xho*I | This study | YajC-FLAG mutant generation |
| pCR-Blunt II-TOPO::Δ*cls*::*aad9* | Δ*cls*::*aad9* cloned between *Bam*HI and *Xho*I | This study | Δ*cls* mutant generation |
| pJL1055::Δ*liaF* | Δ*liaF* cloned between *Bam*HI and *Xho*I | This study | Δ*liaF* mutant generation |
| pJL1055::Δ*liaS::aad9* | Δ*liaS*::*aad9* cloned between *Bam*HI and *Xho*I | This study | Δ*liaS* mutant generation |
| pJL1055::Δ*liaR::aad9* | Δ*liaR::aad9* cloned between *Bam*HI and *Xho*I | This study | Δ*liaR* mutant generation |
| pJL1055::Δ*ropB::aph* | Δ*ropB::aph* cloned between *Bam*HI and *Xho*I | This study | Δ*ropB* mutant generation |
| Δ*cls*/pLZ12Km2::CLS | *cls* and its promotor cloned between BamHI and PstI | This study | CLS expression in GAS |
| Δ*liaF*/pLZ12Km2::LiaF | *liaF* and its promotor cloned between BamHI and PstI | This study | LiaF expression in GAS |
| pJL1055::*liaR-D56A* | *liaR-D56A* cloned between *Bam*HI and *Xho*I | This study | *liaR-D56A* mutant generation |
