# Supplementary material for: ExPortal and the LiaFSR Regulatory System Coordinate the Response to Cell Membrane Stress in Streptococcus pyogenes
Source: mBio. 2020 Sep 15;11(5):e01804-20. doi: 10.1128/mBio.01804-20 (PMC7492735; doi:10.1128/mBio.01804-20)
Supplement: TABLE S3 [file mBio.01804-20-st003.docx]

**Table S3**. Oligonucleotides used in this study.

| Name | Sequence (5' to 3') | Use |
| --- | --- | --- |
| HtrAsfGFP1_F | GATTTGACTAAAACGCAGGGTGGAGGCCGTAAGGGCGAAGAACTGTTTAC | HtrAsfGFP3_R for overlap PCR to fuse sfGFP to HtrA |
| HtrAsfGFP1_R | CCTTACGGCCTCCACCCTGCGTTTTAGTCAAATCTTG | HtrA351BamHI_F for overlap PCR to fuse sfGFP to HtrA |
| HtrAsfGFP3_R | GTATTTAGGTAGCTTGTTTATTTATACAGTTCGTCC | HtrAsfGFP1_F for overlap PCR to fuse sfGFP to HtrA |
| HtrAsfGFP3_F | GGACGAACTGTATAAATAAACAAGCTACCTAAATACTATTAC | HtrA352XhoI_R for overlap PCR to fuse sfGFP to HtrA |
| HtrA351BamHI_F | CGCGGATCCCTAATGGTGAAGTTATCAGACCTG | HtrAsfGFP1_R for overlap PCR to fuse sfGFP to HtrA |
| HtrA352XhoI_R | CCGCTCGAGTGTAGAGATCTTCTTG | HtrAsfGFP3_F for overlap PCR to fuse sfGFP to HtrA |
| LiaSsfGFP1_F | CTTTTATCTTACTCATCTTTATACAGTTCGTCCATACC | LiaSsfGFP3_R for overlap PCR to fuse sfGFP to LiaS |
| LiaSsfGFP1_R | GGACGAACTGTATAAAGATGAGTAAGATAAAAGTGATATTG | LiaS500BamHI_F for overlap PCR to fuse sfGFP to LiaS |
| LiaSsfGFP3_R | GAGGCCGTAAGGGCGAAGAACTGTTTACGGGCGTG | LiaSsfGFP1_F for overlap PCR to fuse sfGFP to LiaS |
| LiaSsfGFP3_F | CGCCCTTACGGCCTCCACCGTCATCTCCCTTCACTATC | LiaS500XhoI_R for overlap PCR to fuse sfGFP to LiaS |
| LiaS500BamHI_F | CGCGGATCCGTCTGATTATCATACCCTTTAGC | LiaS500BamHI_F for overlap PCR to fuse sfGFP to LiaS |
| LiaS500XhoI_R | CCGCTCGAGCAATTACAAACACAGTTAACAACG | LiaSsfGFP1_R for overlap PCR to fuse sfGFP to LiaS |
| YajCsfGFP1_F | CCAAACTTTCCCCGCCTTATTTATACAGTTCGTCCATACC | LiaSsfGFP3_R for overlap PCR to fuse sfGFP to YajC |
| YajCsfGFP1_R | GTATAAA TAAGGCGGGGAAAGTTTGGATG | YajC236BamHI_F for overlap PCR to fuse sfGFP to YajC |
| YajCsfGFP3_F | CGCCCTTACGGCCTCCACCATGGCTTTCAATGGCACTGTC | YajC235XhoI_R for overlap PCR to fuse sfGFP to YajC |
| YajC236BamHI_F | CGCGGATCCGCTTTTAATCCAAACATGTTTTTTCC | YajCsfGFP1_R for overlap PCR to fuse sfGFP to YajC |
| YajC235XhoI_R | CCGCTCGAGCGTAACGATTGGTGGCATGTTTGC | YajCsfGFP3_F for overlap PCR to fuse sfGFP to YajC |
| LiaFsfGFP1_F | GTAACGTTTTTTCATCGTTTATACAGTTCGTCCATACC | LiaSsfGFP3_R for overlap PCR to fuse sfGFP to LiaF |
| LiaFsfGFP1_R | GTATAAA CGATGAAAAAACGTTACTATGCTC | LiaF500BamHI_F for overlap PCR to fuse sfGFP to LiaF |
| LiaFsfGFP3_F | CGCCCTTACGGCCTCCACCCCTATCCACCTCAACATTTCC | LiaF500XhoI_R for overlap PCR to fuse sfGFP to LiaF |
| LiaF500BamHI_F | CGCGGATCCGTCCAGTTGTTCCAAACTCATCG | LiaFsfGFP1_R for overlap PCR to fuse sfGFP to LiaF |
| LiaF500XhoI_R | CCGCTCGAGCATGCTTAATCCCTACATTATC | LiaFsfGFP3_F for overlap PCR to fuse sfGFP to LiaF |
| LiaS3FLAG_F | GATCTTTATAATCACCGTCATGGTCTTTGTAGTCGCCTCCACCGTCATCTCCCTTCACTATCGGCAG | LiaS500XhoI_R for overlap PCR to fuse 3☓FLAG to LiaS |
| LiaS3FLAG_R | GACGGTGATTATAAAGATCATGATATCGATTACAAGGATGACGATGACAAGGATGAGTAAGATAAAAGTGATATTG | LiaS500BamHI_F for overlap PCR to fuse 3☓FLAG to LiaS |
| LiaF3FLAG_F | CATGATCTTTATAATCACCGTCATGGTCTTTGTAGTCGCCTCCACCCCTATCCACCTCAACATTTCC | LiaF500XhoI_R for overlap PCR to fuse 3☓FLAG to LiaF |
| LiaF3FLAG_R | GTGATTATAAAGATCATGATATCGATTACAAGGATGACGATGACAAGCGATGAAAAAACGTTACTATGC | LiaF500BamHI_F for overlap PCR to fuse 3☓FLAG to LiaF |
| HtrA3FLAG_F | GATTATAAAGATCATGATATCGATTACAAGGATGACGATGACAAGTAAACAAGCTACCTAAATACTATTAC | HtrA352XhoI_R for overlap PCR to fuse 3☓FLAG to HtrA |
| HtrA3FLAG_R | CATGATCTTTATAATCACCGTCATGGTCTTTGTAGTCGCCTCCACCCTGCGTTTTAGTCAAATCTTG | HtrA351BamHI_F for overlap PCR to fuse 3☓FLAG to HtrA |
| YajC3FLAG_F | CATGATCTTTATAATCACCGTCATGGTCTTTGTAGTCGCCTCCACCATGGCTTTCAATGGCACTGTCAG | YajC235XhoI_R for overlap PCR to fuse 3☓FLAG to YajC |
| YajC3FLAG_R | GTGATTATAAAGATCATGATATCGATTACAAGGATGACGATGACAAGTAAGGCGGGGAAAGTTTGGATG | YajC236BamHI_F for overlap PCR to fuse 3☓FLAG to YajC |
| MSP286 | TCCCATGCGGATCCTTTCATGATC | Pair with MSP287 to generate 5' flanking region for in-frame *liaF* knockout |
| MSP287 | ATGAAAAAATTTCAATTCTTTTTAAATGTTGAGGTGGATAGGCGATGA | Pair with MSP286 to generate 5' flanking region for in-frame *liaF* KO |
| MSP288 | TCATCGCCTATCCACCTCAACATTTAAAAAGAATTGAAATTTTTTCAT | Pair with MSP289 to generate 3' flanking region for in-frame *liaF* knockout |
| MSP289 | AGGAAACTCGAGTGGATCAAAC | Pair with MSP288 to generate 3' flanking region for in-frame *liaF* knockout |
| 2305F | TCTTGTGGAACACCGTACCG | Pair with MSP291 to generate 5' flanking region for *liaS*::*aad9* gene replacement |
| MSP290 | GTCTAATATCCATGGAAACTCCTTATAATTTTTTTAATCTG | Pair with MSP293 to generate *aad9* gene replacement *liaS::aad9* |
| MSP291 | CAGATTAAAAAAATTATAAGGAGTTTCCATGGATATTAGAC | Pair with 2305F to generate 5' flanking region for *liaS*::*aad9* gene replacement |
| MSP292 | ATTTGTTCGTATGTATTCAAGAGCCAAACAAGAGCATAGTAAC | Pair with 2309R to generate 3' flanking region for *liaS*::*aad9* gene replacement |
| MSP293 | GTTACTATGCTCTTGTTTGGCTCTTGAATACATACGAACAAAT | Pair with MSP290 to generate *aad9* gene replacement for *liaS::aad9* |
| 2309R | TGACTGCATCTGCATAACTA | Pair with MSP292 to generate 3' flanking region for *liaS*::*aad9* gene replacement |
| MSP282 | TATTTGGATCCGCATTAGCC | Pair with MSP295 to generate 5' flanking region for *liaR::aad9* gene replacement |
| MSP295 | CAGATTAAAAAAATTATAATTAGTCCCCCAAGACGATAAT | Pair with MSP282 to generate 5' flanking region for *liaR::aad9* gene replacement |
| MSP294 | ATTATCGTCTTGGGGGACTAATTATAATTTTTTTAATCTG | Pair with MSP297 to generate *aad9* gene replacement *liaR::aad9* |
| MSP297 | GTAAGATAAAAGTGATATTGTTGAATACATACGAACAAAT | Pair with MSP294 to generate *aad9* gene replacement *liaR::aad9* |
| MSP296 | ATTTGTTCGTATGTATTCAACAATATCACTTTTATCTTAC | Pair with MSP285 to generate 3' flanking region for *liaR::aad9* gene replacement |
| MSP285 | TTTGCTCGAGGAATCTATTAAG | Pair with MSP296 to generate 3' flanking region for *liaR::aad9* gene replacement |
| MSP347 | ATACCCTATGATATCGCATTAATTATCGTC | Pair with MSP349 to generate aspartate to alanine at position 56 in LiaR, 5' flank |
| MSP348 | TTATAAGGATCCCATTGCTCAGCTTCC | Pair with MSP350 to generate aspartate to alanine at position 56 in LiaR, 5' flank |
| MSP349 | CCTAACTCTGGCATCACTAGAGCCATAACCAAAACATCTGGCTTC | Pair with MSP347 to generate aspartate to alanine at position 56 in LiaR, 3' flank |
| MSP350 | GAAGCCAGATGTTTTGGTTATGGCTCTAGTGATGCCAGAGTTAGG | Pair with MSP348 to generate aspartate to alanine at position 56 in LiaR, 3' flank |
| GASPclsBamHI_F | CGCGGATCCCCTAAAGTTCCAGCTGCCATG | Pair with GASPclsPstI_R to clone cls and its promoter into pLZ12Km2 between BamHI and PstI |
| GASPclsPstI_R | TGCACTGCAGGGGCATTCTTAATAAATAAGATTG | Pair with GASPclsPstI_F to clone cls and its promoter into pLZ12Km2 between BamHI and PstI |
| GASPliaFBamHI_F | CGCGGATCCATCAGAAACTACTGCTCAAACAC | Pair with GASPliaFPstI_R to clone liaF and its promoter into pLZ12Km2 between BamHI and PstI |
| GASPliaFPstI_R | TGCACTGCAGCAACAATAAAATCGAAAACCCTAG | Pair with GASPliaFPstI_F to clone liaF and its promoter into pLZ12Km2 between BamHI and PstI |
| spxA_F | AAGGATGCGCGGGTTATCTT | qRT PCR; Forward primer for *spxA* |
| spxA_R | ATGCCAAAGCTCTCGATTGC | qRT PCR; Reverse primer for *spxA* |
| spxA_P | TCAAATCAATAACCTCACTAACACTT | qRT PCR; Probe for *spxA* |
| tufA_F | CAACTCGTCACTATGCGCACAT | qRT PCR; Forward primer for *tufA* |
| tufA_R | GAGCGGCACCAGTGATCAT | qRT PCR; Reverse primer for *tufA* |
| tufA-P | CTCCAGGACACGCGGACTACGTTAAAAA | qRT PCR; Probe for *tufA* |
